# Localization of ASV Integrase-DNA Contacts by Site-Directed Crosslinking and their Structural Analysis

**DOI:** 10.1371/journal.pone.0027751

**Published:** 2011-12-01

**Authors:** Elena Peletskaya, Mark Andrake, Alla Gustchina, George Merkel, Jerry Alexandratos, Dongwen Zhou, Ravi S. Bojja, Tadashi Satoh, Mikhail Potapov, Alex Kogon, Viktor Potapov, Alexander Wlodawer, Anna Marie Skalka

**Affiliations:** 1 Biolinx LLC, Alburgh, Vermont, United States of America; 2 Fox Chase Cancer Center, Institute for Cancer Research, Philadelphia, Pennsylvania, United States of America; 3 Macromolecular Crystallography Laboratory, Center for Cancer Research, National Cancer Institute, National Institutes of Health, Frederick, Maryland, United States of America; 4 Shemyakin-Ovchinnikov Institute of Bioorganic Chemistry, Russian Academy of Sciences, Moscow, Russia; Louisiana State University Health Sciences Center, United States of America

## Abstract

**Background:**

We applied crosslinking techniques as a first step in preparation of stable avian sarcoma virus (ASV) integrase (IN)-DNA complexes for crystallographic investigations. These results were then compared with the crystal structures of the prototype foamy virus (PFV) intasome and with published data for other retroviral IN proteins.

**Methodology/Results:**

Photoaffinity crosslinking and site-directed chemical crosslinking were used to localize the sites of contacts with DNA substrates on the surface of ASV IN. Sulfhydryl groups of cysteines engineered into ASV IN and amino-modified nucleotides in DNA substrates were used for attachment of photocrosslinkers. Analysis of photocrosslinking data revealed several specific DNA-protein contacts. To confirm contact sites, thiol-modified nucleotides were introduced into oligo-DNA substrates at suggested points of contact and chemically crosslinked to the cysteines via formation of disulfide bridges. Cysteines incorporated in positions 124 and 146 in the ASV IN core domain were shown to interact directly with host and viral portions of the Y-mer DNA substrate, respectively. Crosslinking of an R244C ASV IN derivative identified contacts at positions 11 and 12 on both strands of viral DNA. The most efficient disulfide crosslinking was observed for complexes of the ASV IN E157C and D64C derivatives with linear viral DNA substrate carrying a thiol-modified scissile phosphate.

**Conclusion:**

Analysis of our crosslinking results as well as published results of retroviral IN protein from other laboratories shows good agreement with the structure of PFV IN and derived ASV, HIV, and MuLV models for the core domain, but only partial agreement for the N- and C-terminal domains. These differences might be explained by structural variations and evolutionary selection for residues at alternate positions to perform analogous functions, and by methodological differences: i.e., a static picture of a particular assembly from crystallography vs. a variety of interactions that might occur during formation of functional IN complexes in solution.

## Introduction

Retroviruses utilize the viral enzyme integrase (IN) for inserting DNA copies of their genomic RNA into host DNA. As this step is necessary for replication of pathogenic retroviruses such as HIV, integrase inhibitors are being developed as an important class of AIDS drugs [1]–[5]. Detailed structural data concerning IN-substrate interactions can contribute greatly to such efforts. Recent success in determining the structure of complexes of prototype foamy virus (PFV) IN with both the viral and target DNAs [6] has provided the foundation for a valuable HIV IN model [7]; however, experimental data for DNA complexes of HIV IN or other integrases from more closely related viruses are still lacking [2]. On the other hand, a large number of structures have been published for the three domains of IN [N-terminal (NTD), core (CCD), and C-terminal (CTD)] from HIV, ASV and other retroviruses, either singly or in pairs [2]. Retroviral integrase is known to be a conformationally dynamic protein and current evidence indicates that it is capable of adopting a defined and/or active conformation only upon binding to its DNA substrate(s) and metal cofactors [8], [9]. Several models of IN-DNA complexes have been developed, originally based on other transposase-DNA structures [10], [11], and more recently on the structures of PFV IN [7], [12], but the conformational variability of integrase, particularly within the inter-domain linkers, exacerbated by the significant differences in their lengths in various viruses, makes such modeling efforts challenging. As detailed structural data on IN-DNA interactions are required to elucidate the molecular mechanism of catalysis and to facilitate drug development efforts, further studies of such complexes remain an imperative. Here we report the use of photoaffinity and chemical crosslinking methods to obtain insight into the interactions of avian sarcoma virus (ASV) IN with its DNA substrates.

Photoaffinity crosslinking and chemical crosslinking are essentially methods of measuring distances between points of interest in macromolecular complexes. By use of reagents with differing linker lengths it is possible to estimate the shortest distance between two sites on a protein or a protein complex. In photoaffinity crosslinking, a heterobifunctional reagent carries one functional group for chemical attachment to a specific target residue in a protein or nucleic acid molecule, and one photoactivatable group that can be triggered by mild UV irradiation into high reactivity, forming a covalent bond with the closest neighbor in a pre-formed complex.

Chemical crosslinking between DNA and target protein involves engineering of sulfhydryl groups into specific positions in the DNA, with the aim of forming disulfide bridges with the cysteine residues in the protein. The positioning of modified nucleotides to enable such chemical crosslinking relies on detailed knowledge of the most likely structure of the complexes. Crosslinking between two thiol groups through formation of the S-S bond serves as confirmation of their close proximity in the complex. However, if successfully prepared, chemically crosslinked protein-DNA complexes not only provide additional validation of the putative contact sites, but such complexes can be further purified in amounts sufficient for other structural studies.

Both of these approaches have been applied previously in studies of IN-DNA complexes, primarily with the HIV-1 IN protein. The feasibility of studying IN-DNA interactions using photoaffinity crosslinking was established in previous investigations in which DNA was modified with halogenated nucleoside-based photocrosslinking agents (I-dU, I-dC) [13], [14] or azidophenacyl group attached to phosphorothioate-modified DNA oligonucleotides [15], [16]. These studies have revealed several important features of HIV-1 IN-DNA binding. Determinants for recognition of viral DNA ends and for joining targets have been mapped to the CCD and CTD of HIV-1 IN. Most of previous studies were focused on HIV-1 IN and they were performed with crosslinking reagents attached to DNA [13]–[16]. The interaction sites were determined by mass-spectrometry and amino acid analysis after proteolytic digestion of the HIV-1 IN [14], [15]. Because these detection methods require relatively large quantities of crosslinked material and their accuracy depends on protein composition, only crosslinks to major peptides can be detected and, in most cases, without amino acid localization.

In contrast, our experimental approach was designed to attach photoactivatable reagents at specified positions within IN for crosslinking to DNA substrates, as well as to utilize the more soluble ASV IN. Application of Cel 1 endonuclease then allowed for single nucleotide localization of the crosslinks. In one set of experiments described in this report, cysteine residues, either normally present or substituted at various positions in IN, have been used as attachment sites for carbene- and nitrene-generating photoreagents [17], [18], whereas DNA was not modified beyond incorporation of radioactive markers. In the second set of experiments, a shorter, amino group-targeted carbene-generating photoreagent was attached to the positions on DNA identified in the first set, and modified DNA was crosslinked to wild type IN, in order to narrow down the most probable points of contact. Finally, in the third set of experiments sulfhydryl groups were engineered into the identified most probable contact positions on DNA, with the aim of forming disulfide bridges with the cysteine residues in the protein. Formation of such bridges under mild conditions at high yields served as the most accurate confirmation of the discovered contacts. These results provide new information about the preferred sites of interaction within the ASV IN-DNA complex. This information is compared with published data on retroviral IN-DNA contacts obtained by the use of the same or other techniques, and the combined set has been compared with IN-DNA interactions observed in crystal structures of PFV IN-DNA complexes.

## Results and Discussion

Photocrosslinking and chemical crosslinking techniques have been used in this study to map IN-DNA contacts with various substrates. Because photoactivatable reagents are fairly bulky, their introduction at or near the assumed sites of protein-DNA contact imposes a limit on distance resolution by this approach. Usually, multiple crosslinks are detected, dependent on spatial restrictions at a particular protein/DNA interface and the flexibility of the linker, on activated photocrosslinker preferences for certain chemistries of target groups, on general movements of the components of biomolecular complex, etc. To achieve higher resolution of localization of contact sites we employed three-step crosslinking. We first identified the nucleotides that were crosslinked by a long-linker photoactivatable reagent placed at selected positions in the ASV IN protein. In the second step, a short-linker photoreagent was placed at the most promising positions identified on DNA and crosslinked to IN protein for more accurate contact localization. Finally, the localization results of these two steps were refined by near-zero-length chemical crosslinking between unique cysteines on IN and unique SH-modified nucleotides on DNA substrates to confirm the positions of IN-DNA contacts.

### Design of DNA substrates

**Figure 1:**
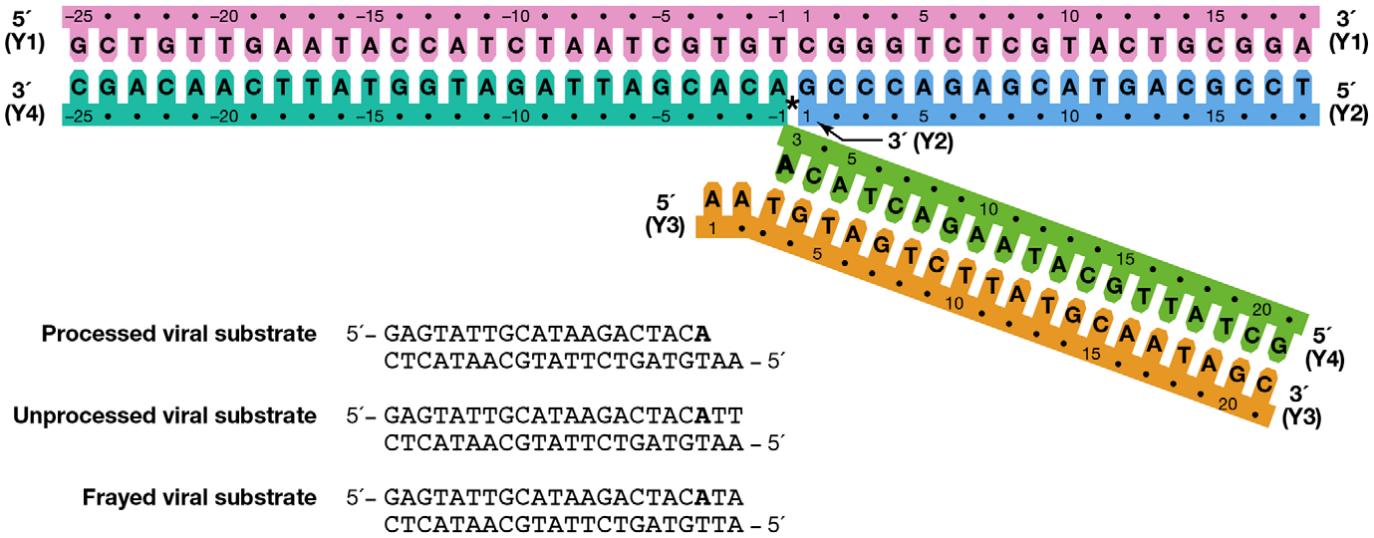
DNA substrates used for crosslinking to ASV IN. The indicated numbering is as used throughout the manuscript. The Y-mer strand Y4 has non-wild type bases at positions 20 and 21 in order to increase the stability of this arm of the substrate. DNA strands in the Y-mer substrate are colored for identification as in **Figures 3** **–** [Fig pone-0027751-g004] [Fig pone-0027751-g005] **6**. The host portion of strand Y4 is colored in lighter shade of blue to distinguish the host sequence from the viral sequence after covalent joining to viral DNA. In linear and Ymer DNAs, the conserved adenine preceding the scissile phosphate is shown in bold.

In order to study different stages of the integration process, viral linear and Y-mer DNA substrates were employed to mimic the intermediate steps of processing viral DNA and joining the viral DNA substrate to host DNA. Specifically, blunt-end, unpaired end, and processed linear DNA substrates represented unprocessed, frayed, and cleaved U3 LTR viral end DNA, respectively (**Figure 1**). Y-mer substrates represent an integration intermediate in which one strand of a viral DNA end is joined to the host DNA (also known as a half-site strand-transfer intermediate) (**Figure 1**).

For the different crosslinking experiments, several modified DNA substrates were used: a) unmodified DNA, when a photoactivatable moiety was engineered into IN molecule; b) DNA with selected thymidines replaced by anchor 5-aminouridine residues for further attachment of amino-specific photocrosslinking reagent to crosslink to the IN molecule; c) DNA with selected adenosines and guanidines replaced by their corresponding 7-thio-derivatives in the mixed disulfide activated form (see **Methods S1**) for chemical crosslinking with target cysteine on the IN molecule.

In the discussion below, the nucleotide positions in both strands of the viral end substrate are numbered from the blunt end that contains the conservative CA dinucleotide preceding the scissile phosphate. This numbering is maintained in the viral end portion of the integration intermediate Y-mer substrate, so that the processed strand nucleotide that is the closest to the junction of the integration site is assigned #3. The first nucleotide position in the viral 5′ overhang of the non-cleaved strand remains #1 (**Figure 1**, orange strand). For the host (target) portion of the Y-mer substrate the nucleotide numbering in both strands starts from the junction of the integration site (See **Figure 1**, pink and blue strands).

### Design of Cys derivatives of ASV IN

**Figure 2:**
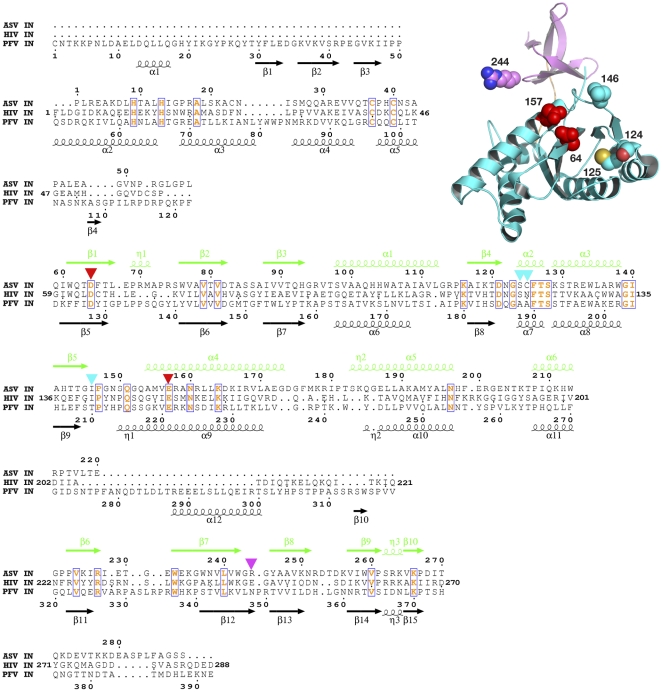
Structure-based sequence alignment of full-length ASV, HIV-1, and PFV IN proteins. ASV IN numbering is shown above the sequences and the structural elements are marked in green; PFV IN numbering and structural elements (black) are shown below. Numbering for HIV-1 IN is shown at the beginning and end of the lines only. The conserved amino acids, including the catalytic ASV IN residues Asp121 and Glu157, are red and boxed. Triangles mark residues that were changed to cysteines in ASV IN: red for the amino acids in the active site, cyan for other residues in the CCD, and magenta for the amino acids in the CTD. The structure of the ASV IN CCD and CTD (PDB code 1COM) with the location of the introduced cysteines is shown in the upper right corner, with the colors corresponding to the scheme described above.

Several IN derivatives with cysteine residues positioned at the putative points of contact with DNA substrates (**Figures 2** **,** **3** **,** **4** **,** **5** **,** **6**) were created by site-directed mutagenesis (**Table 1**). These cysteines were employed as “anchor” amino acids for attachment of the thiol-specific photoactivatable reagents. A single cysteine residue in the wild-type core domain of ASV IN (Cys125) was retained in some of the proteins, or replaced by serine in others. Positions 64, 124, 146, 157, and 244 were selected for substitution with cysteine, as follows:

**Figure 3:**
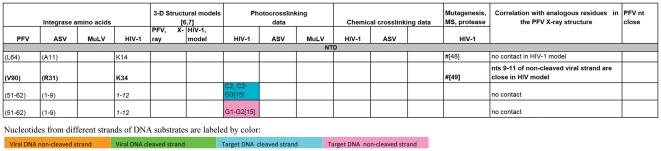
A comparison of all the IN-DNA contact points that have been determined experimentally or modeled for the NTD IN. The amino acid residues are presented in black, unless a particular residue comes from specified IN monomer in PFV intasome structure as in Refs. [6], [7]. Specific residues shown to interact with DNA that are either in good correlation with the PFV structural results or do not contradict them are bolded. # -this amino acid is in contact with DNA, but the nucleotide is not determined. (G377) - The amino acid residues in parentheses indicate structural analogs to the ones implicated in DNA binding by experimental data. Nucleotides from different strands of DNA substrates are labeled by colors corresponding to the scheme used in **Figure 1** and noted above. **“G5{15}” -** In this example and throughout Figures 3, 4, 5, 6 the nucleotide numbers correspond to the numbering scheme shown in **Figure 1**. The numbers in the curly brackets are as in the structure of PFV IN and the model of HIV-1 IN [6], [7]. If listed, the letter designating a nucleotide comes from the original data. All reported contacts are references to original publications with numbers in brackets; our data are marked with asterisks (e.g. A3*).

**Figure 4 pone-0027751-g004:**
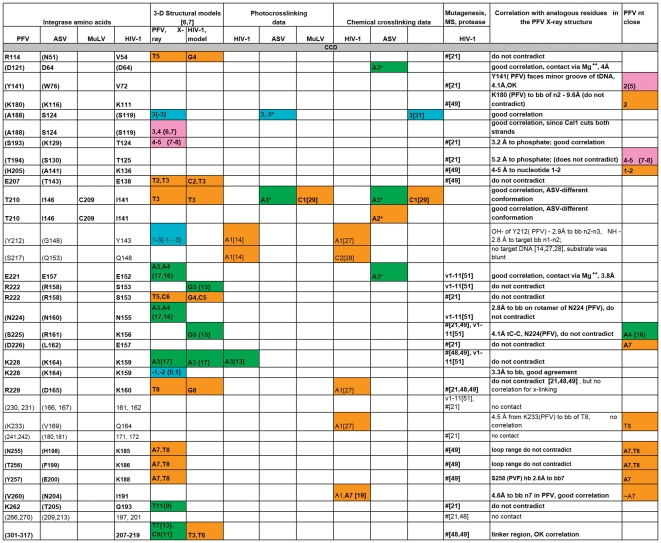
A comparison of all the IN-DNA contact points that have been determined experimentally or modeled for the CCD IN. For details, see legend to Figure 3.

**Figure 5 pone-0027751-g005:**
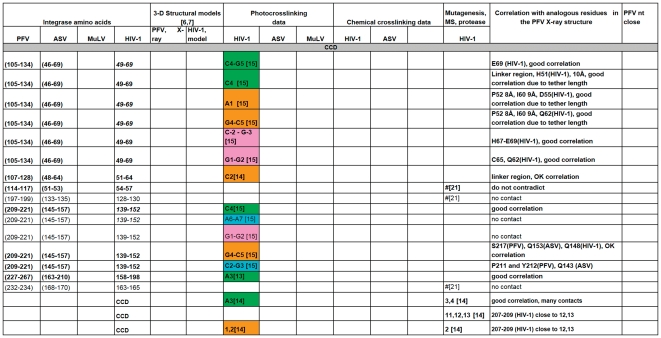
A comparison of all the IN-DNA contact points that have been determined experimentally or modeled for the CCD IN (continued). For details, see legend to Figure 3.

**Figure 6:**
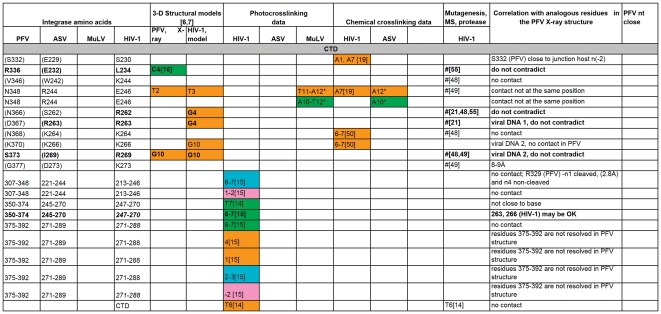
A comparison of all the IN-DNA contact points that have been determined experimentally or modeled for the CTD IN. For details, see legend to Figure 3.

**Table 1:** Substitutions in the ASV IN derivatives and their enzymatic activities (crosslinkers placed at residues that are bold).

Protein	% WT processing^1^	% WT joining^2^
Wild-type	100	100
F199K	238	102
C125S, **I146C**, F199K	80	72
C125S, F199K, **R244C**	<1	<1
F199K, **R244C**	<5–12	<1
C125S, **I146C**, F199K,**R244C**	4	2
**I146C**, F199K,**R244C**	**<1**	<1
**D64C**, F199K	4	20
**E157C**, F199K	6	1
C23S, C125S, **E157C** F199K	ND	<4
C23S, C125S, **E157C** F199K,W259A	<4	<4
**S124C**, C125S, F199K	38	16

The dominant **Cys** for crosslinking is **bolded**.

1 Processing of viral ends as assayed either by a fluorescence anisotropy release assay or a radioactively labeled processing assay in 10 mM Mg^2+^ for 30 min.

2 Single end joining assessed by a fluorescent joining assay [33].

R244C substitution severely compromises activity in all IN derivatives that contain it.

The active site residues Asp64 and Glu157 were obvious choices for substitution with Cys due to their functional close contact with the DNA substrate. The other putative contact positions in the ASV IN-DNA complex were predicted based on crosslinking data [13], [15], [16], [19], mutagenesis studies [20]–[22], and structure-based multiple sequence alignments involving analysis of superimposed 3D structures of individual and two-domain constructs of IN proteins (PDB codes 1EX4 [23], 1C6V [24], 1C0M [25], and 1K6Y [26], Mu transposase (PDB code 1BCO [10]), and the Tn5 transposase DNA complex (PDB code 1F3I [11].

The choice to substitute Cys for Ile146, located in the flexible loop near the active site of ASV IN, was based on the results from a number of previous studies. Chemical crosslinking and photocrosslinking experiments [13], [14], [27]–[29] have suggested that the flexible loop near the IN active site is likely to make contact with the 5′-end of the non-processed viral DNA. Results of Esposito and Craigie [14] in which 5-iododeoxyuracil was placed at the 5′-end of HIV-1 non-processed DNA demonstrated a high efficiency (∼10%) of photocrosslinking to residues Tyr143 and Gln148 in the flexible loop of HIV-1 IN. Q148C was also reported to chemically crosslink to thiol-modified 5′-end of viral DNA [27]. Johnson *et al.* [28] reported the formation of S-S bond between Y143C and position 2 next to 5′-end of the non-processed viral DNA. Similar experiments with murine leukemia virus (MuLV) IN [29] implicated Cys209 as another possible point of contact for the cognate 5′-end. When aligned using the program CLUSTALW, the positions corresponding to MuLV IN residue 209 in HIV-1 and ASV IN are Ile141 and Ile146, respectively (**Figure 2**). These residues are located within the flexible loop region (amino acids 141–149), adjacent to the active site in the core domain of IN. Consequently, to establish covalent links to the end of the DNA substrate near the IN active site, we replaced Ile146 with cysteine (**Table 1**).

Although retroviral DNA can be inserted by IN into almost any site in cellular DNA, limited target site preferences have been described both *in vitro* and *in vivo*. Katzman and co-workers screened HIV-1 infected patient derived integrase sequences for amino acid changes in the catalytic core of HIV-1 IN and identified Ser119 as contributing to target site preferences [30], as assayed by integrase joining *in vitro*. These researchers were able to extend their findings to the integrases of a non-primate lentivirus Visna and the more distantly related alpharetrovirus, ASV [31]. Selection of target DNA sites is therefore likely to be a general property of the analogous residue in most retroviral integrases. Indeed, the corresponding residue in PFV is intimately involved in target DNA binding [6]. Non-conservative amino acid substitutions at this position in all three integrases exhibited a phenotype in which the processing activity was unaffected but the joining activity was significantly compromised, and it was hypothesized that this amino acid may be a critical component of the cellular DNA binding site on integrase proteins. To test this idea, we placed a photocrosslinker-anchoring cysteine residue at the analogous position in ASV IN, Ser124 (**Figure 2**).

Finally, Gao *et al.* [19] postulated that the C-terminal domain of HIV-1 IN makes most efficient contact with position +7 on the “non-cleaved” strand of viral DNA. The preferred site of interaction was identified as Glu246 of C-terminal domain of HIV-1 IN. Consequently, the corresponding ASV IN residue, Arg244, was replaced by a cysteine (**Figure 2** and **Table 1**).

### Photocrosslinking of modified IN to DNA substrates

#### Modification of IN derivatives by photocrosslinkers

In the initial experiments, photocrosslinking to DNA substrates was performed using wild type ASV IN and the cysteine substituted derivatives described above and listed in **Table 1**. These proteins were modified at one or two cysteine positions by coupling with photoactivatable thiol-specific compounds, either N-bromoacetyl-N′-{2,3-dihydroxy-3-[3-(3-(trifluoromethyl)diazirin-3-yl)phenyl]propionyl}-ethylenediamine (BATDHP) [17], [18], or azidophenacylthiopyridine (APTP) [32] (see **Methods S1**).

Because the N-terminal domain (NTD) of ASV IN contains three cysteine residues that are either involved in Zn^2+^ coordination (Cys38, Cys40) or structurally important (Cys23), reaction conditions were found that favored modification of the newly introduced solvent accessible cysteines and left those in the NTD unmodified. These conditions were found empirically by varying pH, temperature, and time of the reaction. The presence of the photocrosslinking moiety at selected positions and its absence in the NTD was confirmed by MALDI-TOF mass-spectroscopy of tryptic peptides obtained from IN-DNA adducts excised from gels (data not shown).

Apart from the activity changes due to Cys substitutions, the introduction of photocrosslinkers did not result in significant changes in protein function, as measured by comparison of the enzymatic activities of the modified and non-modified IN proteins, using a standard disintegration assay [33] (**Figure S1A** – schematics; **Figure S1B** – results).

The IN-DNA complexes prepared from the modified IN derivatives and oligonucleotide substrates were irradiated with long-wavelength UV-light, to activate the photocrosslinkers and produce covalent links between the IN protein and DNA. The products were then separated by gel electrophoresis, visualized, and quantified with a PhosphorImager (see Photocrosslinking in Materials and Methods).

### Procedures for localization of photocrosslinks

**Figure 7:**
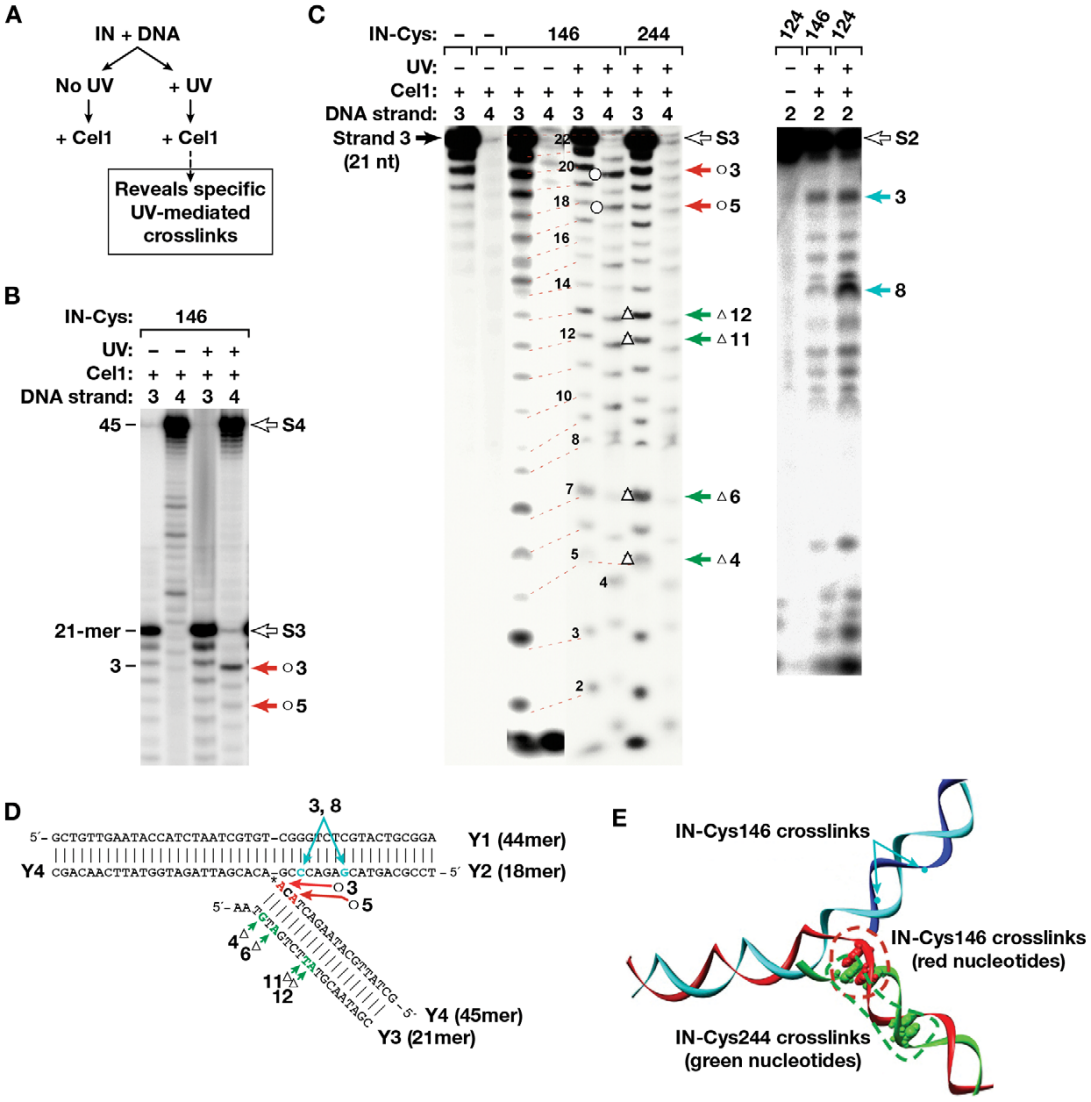
Cel 1-based localization of the crosslinking sites on the Y-mer DNA. (A) The method for detecting specific UV-mediated crosslinks in Ymer DNA is outlined. (B) Cel 1 cleavage of the photocrosslinked complex of IN I146C. Products from Y-mer DNAs labeled at the 5′-end of strand 3 or 4 (marked above each lane) are shown. The filled arrows point to prominent Cel 1 products, indicative of a bulky adduct at the conserved viral CA dinucleotide in strand 4. Open arrows mark the position of non-cleaved substrate strands. (C) Cel 1 cleavage of various photocrosslinked complexes of Cys-modified derivatives of IN with Y-mer DNA labeled at strands 2, 3, or 4. Numbers above the gels indicate which DNA strand in the Y-mer was labeled. Open arrows mark the position of non-cleaved substrate strands. Numbers to the left of the gel indicate length in nucleotides, and arrows to the right mark the positions of adducts of IN with DNA. In both B and C, products were separated by denaturing gel electrophoresis and then visualized with a PhosphorImager. (D) Y-mer DNA sequence with positions of preferred crosslinking detected by Cel1 indicated for each IN derivative by red (I146C), green (R244C) and teal (S124C) arrows; (E) 3-D model of the Y-mer DNA with positions of preferred crosslinking detected by Cel1 indicated for each IN derivative by green (C244), red (C146) and teal (C124) dots.

To detect the preferred sites of IN photocrosslinking on the DNA substrates, a photoactivatable reagent with a *cis*-diol bond in the linker, BATDHP, was specifically cleaved by mild periodate treatment. This resulted in the transfer of the bulky aromatic part of the photocrosslinking reagent from IN to the DNA, thereby producing a cleavage site for the endonuclease Cel1 (Transgenomics, Inc. [34]–[36]), which cuts double-stranded DNA at mispaired bases or sites with bulky nucleotide adducts essentially reporting their location (**Figure 7A**). Because Cel1 cleaves both DNA strands at such sites, a separate set of experiments was performed in which each of the four strands that constituted the Y-mer (labeled 1–4 as in **Figure 1**) were separately labeled with ^32^P at the 5′-end. The crosslinked strands were then identified after denaturing polyacrylamide gel electrophoresis by their reduced mobility due to the covalent attachment of IN (**Figure S2**). As Cel1 occupies about a 10-bp stretch of the DNA substrate and requires substrates longer than 20 bp to process consistently, this approach was only used for detection of photocrosslinks on the Y-mer DNA and not on the shorter linear substrates. Because most of the crosslinks to the Y-mer DNA mapped to the viral portion, these results were combined with crosslink locations identified in the linear substrates and, together with the data published by others, were used to select positions on linear DNA substrates for placement of photocrosslinking reagents and chemical crosslinking moieties.

### Proximities identified from crosslinking IN residues to the DNA substrates

WT IN (containing Cys125 as the only cysteine available for modification with photocrosslinking reagents) was used as a negative control in all our crosslinking experiments. As that position was not expected to be in contact with the DNA substrate, it is not surprising that no significant photocrosslinking was observed with WT IN (see **Fig. S2** and **Results S1**). Strand 4 on the Y-mer (see also **Figure 1**, green strand) was found to be the most likely target for crosslinking for modified IN derivatives with Cys residues at positions 146, 244, and 146 plus 244 (**Figures S2**, **S3** and **Results S1**). This strand of DNA is analogous to the newly joined viral DNA strand. Photocrosslinking from Cys124 resulted in covalent binding to the host portion of the Y-mer substrate, specifically 3 and 8 nucleotides away from the integration junction (**Figure 7C–E**). These IN-DNA contacts are in good agreement with the suggested role of ASV IN residue Ser124 in host site binding/selectivity [31].

Photocrosslinking from Cys146 resulted in covalent binding to the viral portion of strand Y4 (**Figure 7B–E**), primarily one nucleotide to the 5′ side of the scissile phosphate (position 3). Analysis of phenol/chloroform-separated covalent complexes of IN-DNA also showed interactions at position 3 of this strand in a linear substrate (L3). Cel1 cleavage of photocrosslinked products obtained with the Cys244 derivative uncovered a range of sites predominantly around positions 9–12 in Y4; 7–10 and 12 in Y3 (**Figure 7C–E**). Such variability may be due to mobility of the CTD. The results of these and additional experiments with ASV IN derivatives are summarized in **Table 2**.

**Table 2:** Preferred position for photocrosslinking of modified IN Cys derivatives to a Y-mer DNA substrate.

ASV IN derivative and DNA type	Cys146		Cys146,244		Cys244		Cys124	
Cel 1 reaction substrate	Y4	Y3	Y4	Y3	Y4	Y3	Y1	Y2
**Untreated crosslinking reaction**	5,**3**	12	5,**3**,1t	**3**	**10,11**,1t	6,**12**,16		**3t**,8t
**NaIO_4_− treated crosslinking reaction**	**3**	**3**,11,12,		**3**	3–5, 1t	**8–10**, 18		**3t**,8t
**Phenol fraction treated with NaIO_4_**	9,**3**	**3**, 7, 8	**11**		**9–12**	1–4, 7	4t*	**3t**,8t

Bold letters stand for the most prominent contacts revealed by Cel 1 endonuclease analysis,

*numbers followed by “t” represent positions in the target DNA portion of the Y-mer substrate.

### Photocrosslinking from specific nucleotides in linear DNA substrates to IN

In order to refine IN-DNA contact localization data for the CTD, we attached a photoactivatable reagent with a shorter linker to selected nucleotides on linear substrates for crosslinking to IN. Three different synthetic DNA substrates were designed with amino-modified nucleotides introduced in positions 8 and 11 of strand L3 and position 12 of strand L4 (see Materials and methods). The amino groups served as specific anchors for DNA modification with the NHS ester of the carbene-generating (N-hydroxysuccinimidyl-3-[3-(trifluoromethyl)diazirin-3-yl]benzoate). The resulting modified DNA oligonucleotides were labeled with ^32^P and annealed to the corresponding complementary oligonucleotide to form 22 bp linear DNA substrates. The highest efficiency of crosslinking to WT IN was found for position 11 on strand L3 and position 12 on strand L4. Efficiency of crosslinking from position 8 of strand L3 was less than half of that for position 11 on strand L3 (**Figure S4**). These data show that these positions are in close contact with IN and together with the results from the previous experiment suggest a contact between nucleotides at positions 11-L3 and 12-L4 of the linear substrate and IN position 244 in the CTD.

### Chemical crosslinking of modified DNA substrates to residues near the active center of ASV IN

Mixed disulfide chemical crosslinking has been used previously to locate points of contact between HIV-1 reverse transcriptase and DNA with better accuracy and to obtain preparative quantities of tethered RT-DNA complexes [37]. The information derived from our photocrosslinking experiments was used to incorporate mixed disulfide-activated thiol-containing nucleotide derivatives at specific positions of synthetic 22 bp DNA oligonucleotides, representing the U3 viral end (see Materials and Methods). In addition, the 5′-end on the non-cleaved strand of viral DNA was chosen for S-S chemical crosslinking because various lines of evidence have indicated that its binding increases the stability of IN-DNA complex [38]–[40]. Double-stranded Y-mer and linear DNA substrates prepared with these oligonucleotides were subjected to chemical crosslinking with each of the cysteine derivatives of ASV IN (**Table 1**). As previous results have indicated that the viral end binding is facilitated by the “breathing” (or fraying) that normally occurs preferentially at DNA termini [41]–[44] most of the linear substrates for S-S crosslinking were prepared with “frayed” ends (**Figure 1**).

Because IN-DNA binding efficiency differs from one IN derivative to another, the crosslinking data can be interpreted only by comparing the crosslinking yields with substrates modified at different nucleotide positions (also see **Results S1**). All analytical experiments were carried out in physiological buffers, at low IN concentrations, with the IN∶DNA ratio reflecting theoretical stoichiometry (2∶1 for linear DNA and 4∶1 for Y-mer DNA). The results of these experiments are summarized in **Table 3**. Representative data are provided in **Figure S5**. Following are our observations with respect to each of the relevant Cys-substituted IN derivatives:

**Table 3:** A comparative summary of IN-DNA S-S crosslinking with mixed disulfide–modified substrates.

*Substrate*	*L4-3*	*L4-10*	*L4-12*	*L3-2*	*Y3-2*	*L3-12*	*Y3-12*	*L4-3 processed*
*Cys position*								
146	**++**				+	+/−		
146, 244	+/−	+/−	++	**++**	**++**			
244	+/−	+/−				+d*	**++**d*	
125	+			+	+			
125, 157	**++**		+/−		+	+		**++**

The efficiency of crosslinking is shown by a number of (+) signs. The most efficient contact sites are shown in bold (**++**). The DNA substrates used are shown in the top row; d* indicates preference for dimer formation. Linear(L) and Y-mer(Y) dsDNA substrates with thiol groups introduced in the bases of certain strand (e.g. L4 carries a modification in strand 4 (processed strand of viral end) at certain position (e.g. L3-12 is modified at 12^th^ nucleotide from 5′-end of the non-processed strand, or L4-3 – at 3^rd^ position (A) of processed strand that is next to scissile phosphate.

#### Cys125

Only low amounts of crosslinking were seen with linear and Y-mer substrates containing a thiol modification near the 5′ nucleotide on strand 3 and the opposite nucleotides on strand 4 (**Figure 1**, orange and green strands, **Figure S5**, **Table 3**). These data were consistent with our photocrosslinking results suggesting no direct contact between Cys125 and viral DNA. While small, the Cys125 contribution to IN-DNA crosslinking was still taken into account in all other chemical crosslinking experiments where Cys125 remained intact.

#### Cys146

The most prominent contact with Cys146 (residing in the flexible loop near the active site in core domain) was observed at the 3′-end nucleotide of the strand L4 (**Table 3**). Significant crosslinking was also detected at positions 1 and 2 of strands L3 and Y3 (**Figure S5** and **Table 3**). These data are in good agreement with our photocrosslinking results and with previously reported involvement of the flexible loop with the viral end of DNA close to scissile phosphate [13], [14], [27]–[29].

#### Cys244

The C-terminal domain Cys244 was found to crosslink with the viral end of DNA at positions 10 of strand 4 or position 12 of strand 3 in both linear and Y-mer oligonucleotides, in agreement with our photocrosslinking data (**Figure S5**, **Table 3**). These contact positions differ from the chemical crosslinking results [19] that placed the homologous amino acid residue 246 of HIV-1 IN in contact with position 7 of the non-cleaved strand of viral DNA. This discrepancy could be attributed to the significant differences in the lengths of the linker regions between the CCD and CTD in HIV-1 IN (18 aa) and ASV IN (7 aa), compared to that in PFV IN (48 aa), possibly resulting in different relative positioning of their CTDs in an intasome.

### Chemical crosslinking of modified DNA substrates to catalytic residues in ASV IN

In order to find the best approach for producing stable IN-DNA complexes for structural studies, we compared the crosslinking efficiencies of several full-length ASV IN derivatives carrying Cys substitutions in the active site, including the metal cofactor binding residues Asp64 or Glu157, and the Cys already present at position 125. The same substitutions were introduced into the core domain which was then expressed separately. In some constructs the Cys125 was substituted with serine, and a W259A substitution was included. The W259A replacement has been shown to block formation of ASV IN dimers [45].

**Figure 8:**
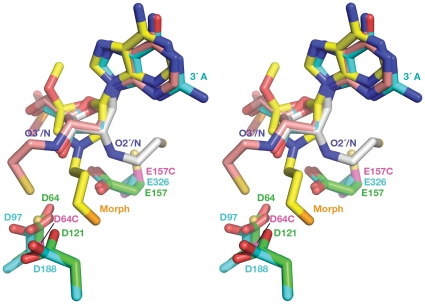
Design of modified 3′-end adenine. A nucleotide at the 3′-end of DNA in TN5 transposase structure is shown in blue, morpholino adenosine analog in yellow, nucleotides with modifications on the C3′ and on C2′ of ribose,are shown in pink and gray respectively. Catalytic residues are shown in blue for transposase and in green for ASV IN. Substituted catalytic residues D64C and E157C are shown in magenta.

The 22-mer dsDNA substrates used in these experiments were designed to represent the processed (recessed) U3 portion of the viral genome and contained modified adenosine in the 3′ position of the processed strand (**Figure 1**). One modified adenosine contained 3-mercaptopropanol phosphodiester at 3′ position of the 3′-terminal desoxyribose; in a second substrate the same desoxyribose was substituted by N-mercaptoethyl derivative of morpholine. To find the optimal position for the thiol group in the nucleotide, the structure of TN5 transposase complexed with Tn5 transposon end DNA (PDB code 1MUH) was used as a reference. Superposition of the active sites of TN5 and core domain of ASV IN (PDB code 1VSH) allowed modeling of the 3′-end nucleotide in the active site of ASV IN (**Figure 8**). Both modified oligonucleotides were designed to present their thiols for direct interaction with a Cys residue introduced in the active site of the ASV IN at the positions of the catalytic residues, Asp64 and Glu157.

The most efficient crosslinking to E157C and D64C was observed in the presence of 10 mM MgCl_2_, indicating that, in contrast to other IN-DNA contact sites, crosslinking to these derivatives required the presence of Mg^2+^ (data not shown). The fact that the E157C IN construct is capable of binding a metal cation suggests that the ion binds in site I (Asp64–Asp121), as seen in previous structures of IN with a disordered region encompassing the Glu157 residue [46], [47]. We also showed in previous experiments with a D64N derivative that Asp121 alone can bind a single Zn^2+^ cation in site I [47]. It is therefore quite plausible that the D64C derivative could likewise coordinate Mg^2+^ with Asp121 in site I alone (or perhaps with Glu157 in site II) in the presence of additional contacts with DNA. Such interactions, in turn, could stabilize the DNA-IN complex at the active site. A crystal structure of an ASV IN-DNA complex is required to confirm this hypothesis.

**Figure 9:**
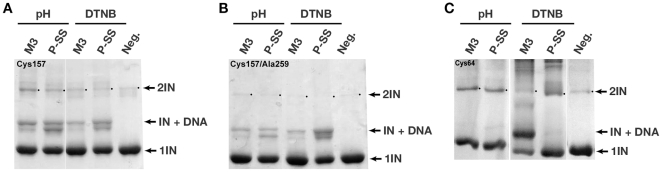
S-S crosslinking of the ASV IN active site derivatives to modified linear dsDNA substrates. Sample Coomassie–stained polyacrylamide gels with IN-DNA crosslinks. pH indicates pH-induced crosslinking; DTNB, DTNB-mediated crosslinking; lanes labeled P-SS correspond to crosslinking to DNA carrying thiol modification on the 3′ phosphate, lanes labeled M3 correspond to crosslinking to DNA carrying thiol modification on the morpholino adenosine analog. The lane marked Neg represents samples with no DNA. 2IN, IN+DNA and 1IN designate dimeric IN bands, adduct bands and monomeric IN bands, respectively. Molecular weight marker lanes are not shown as IN-DNA the monomer (lower) and dimer (upper) bands provide internal calibration. The figure shows the results only for full length INs. Panel A shows crosslinking to the C23S/C125S/E157C/F199K ASV IN derivative (labeled Cys157 after the key mutation); panel B to C23S/C125S/E157C/F199K/W259A (labeled Cys157/Ala259), and panel C to D64C/F199K (labeled Cys64).

All active site substituted derivatives were subjected to pH-dependent and DTNB-mediated protocols to promote formation of S-S bonds with the DNA substrates, and the results are summarized in **Figure 9**. For experiments performed with the full length E157C IN, the highest yields were observed with the 3′-attached 3-mercaptopropanol phosphodiester modified substrates (P-SS), similar for both pH and DTNB activation. The C23S/C125S/E157C/F199K IN derivative (**Figure 9A**) produced higher yields of crosslinking than the single E157C IN derivative (not shown) with both modified DNA substrates, regardless of the activation method (pH-dependent or DTNB). Crosslinking of the C23S/C125S/E157C/F199K/W259A IN derivative with both modified DNA substrates using the pH activation method produced slightly lower yields than crosslinking of the C23S/C125S/E157C/F199K IN derivative (Compare **Figure 9A and 9B** pH-P-SS lanes), and no adduct band was observed above the position of dimeric IN in **Figure 9B**. Protein migrating at the 2IN position and weak bands above this on SDS PAGE represent covalently linked IN dimers and IN dimers linked to DNA, respectively. Because the W259A substitution has been shown to impair dimer formation [45], this result was anticipated. However, even if the majority of IN was dimeric in complex with DNA (i.e. W259 containing protein in **Figure 9A**), the predominant adduct band is expected to migrate in an SDS gel as a monomer+DNA adduct, as crosslinks between IN proteins are unlikely with this experimental design.

After the structure of the PFV intasome became available we verified that the position of the 3′nucleotide in the active site of TN5 transposase is similar to its counterpart in PFV IN. Although the orientation of the 3′- end nucleotide is slightly different in PFV IN, the presence of the flexible linkers carrying thiol groups is likely to have allowed successful crosslinking of both modified nucleotides to ASV IN D64C and E157C derivatives. The requirement for metal ions for the efficient crosslinking of Cys derivatives to substrates containing thiol at the 3′-end of the processed strand indicates that binding to the viral DNA substrate is preserved upon replacement of one of the catalytic residues of ASV IN with Cys.

### Rationalization of the crosslinking data in the context of currently available structural information

Photocrosslinking and chemical crosslinking data available to date, combined with results presented in this study, were compared with the interactions observed in the recently solved structures of the PFV intasome. In order to identify corresponding residues, a structure-based sequence alignment of ASV IN, HIV-1 IN, and PFV IN was created by superimposing the coordinates of the individual domains of the ASV and HIV-1 INs on the structure of full-length PFV IN complexed with the viral and target DNAs (**Figure 2**). A summary of our analyses is presented in **Figures 3** **,** **4** **,** **5** **,** **6**. Comparison of the data from different sources was complicated by the fact that different ways of numbering of the nucleotides in the DNA substrates have been used by various investigators. For example, in several studies numbering of the cleaved strand starts with the first adenine on the 3′-end, resulting in the assigning of the numbers “−1” and “−2” to the two extra nucleotides on the 5′-end of the non-cleaved strand, (i.e. Gao et al. [19]). In the structures of PFV IN complexed with DNA, numbering from the 5′-end was introduced for the cleaved strand of viral DNA, placing the 3′- end adenine under number 17. Because the length of the oligonucleotides used in different studies varies, numbering from the 5′-end introduces additional confusion, as the number designations for the structurally equivalent nucleotides in the cleaved strands of different length would be different. We, as well as some others, elected to number the non-cleaved viral DNA strand from the first nucleotide at the 5′-end. The first nucleotide on the 3′- end of the cleaved strand of processed substrate (closest to the junction in Y-mer or X-mer integration intermediate substrate) is assigned #3 (**Figure 1**, green strand). For the target DNA, numbering of both strands starts from the junction of the integration site (**Figure 1**, pink and blue strands). In order to compare our crosslinking results with IN-DNA contact data from other laboratories, we have translated all nucleotide numbering of the strands that vary in substrate DNAs into this format. However, as a reference, we have included in curly brackets the original numbers from Maertens et al. [6] and Krishnan et al. [7] for the nucleotides shown to interact with PFV IN.

To identify the functionally equivalent residues in ASV, HIV, and PFV INs, the structures of individual NTD, CCD, and CTD domains were superimposed upon the structure of the complex of PFV IN with DNA (PDB code 3OS0). Some chemical and photocrosslinking data identify the individual points of contact between the proteins and DNA. If a method does not allow one to specify a single contact point in both protein and DNA, then these data are not sufficient to establish the exact correlation with results from crystallography, even when they do not contradict them. Such data can be categorized only as either “do not contradict,” if IN and DNA are in proximity to each other in the PFV IN structure, or “no contact,” if IN is remote from substrate DNA in the PFV intasome. Specific residues shown to interact with DNA that are either in good correlation with the PFV structural results or do not contradict them are bolded in **Figures 3** **,** **4** **,** **5** **,** **6**. The tabulated results show that the correlation between the PFV crystal structures and experimental data from crosslinking, mutagenesis, protease mapping, and mass spectrometry for ASV, MuLV, and HIV-1 IN proteins is highest for the CCD (**Figures 4** **,** **5** with color coding as in **Figure 1**). The crosslinking results that pinpoint individual IN-DNA contacts in the NTD and CTD of HIV-1 and ASV IN proteins show low correlation with the interactions observed in the structure of PFV IN complexed with DNA.

#### Interactions between DNA and the NTD

Very limited crosslinking data are available for the NTD. Both subdomains of the NTD of PFV (NED and the conserved subdomain) interact with viral DNA in the PFV intasome crystal. There is no target DNA in the proximity of the NTD of PFV, and no viral DNA in the proximity to the region analogous to the HIV-1 NTD peptide (amino acids 1–12) reported to interact with viral DNA by Heuer et al. [15], [16]. Two HIV-1 IN amino acids in the NTD, Lys14 and Lys34, were implicated as having contacts with DNA by mass spectrometry [48] and proteolysis mapping [49], respectively, but only Lys34 (Val90 in PFV) appears to be relatively close (∼9 Å) to nucleotides 9–11 of the non-cleaved viral strand in the PFV IN structure, whereas Lys14 lies more than 20 Å away from the DNA. It should be noted that only two of the four PFV NTDs are visible in the intasome crystal complexes, and alternate NTD positions for these unseen NTDs could account for proximity data for various solution experiments.

#### Interactions between DNA and the CTD

**Figure 10:**
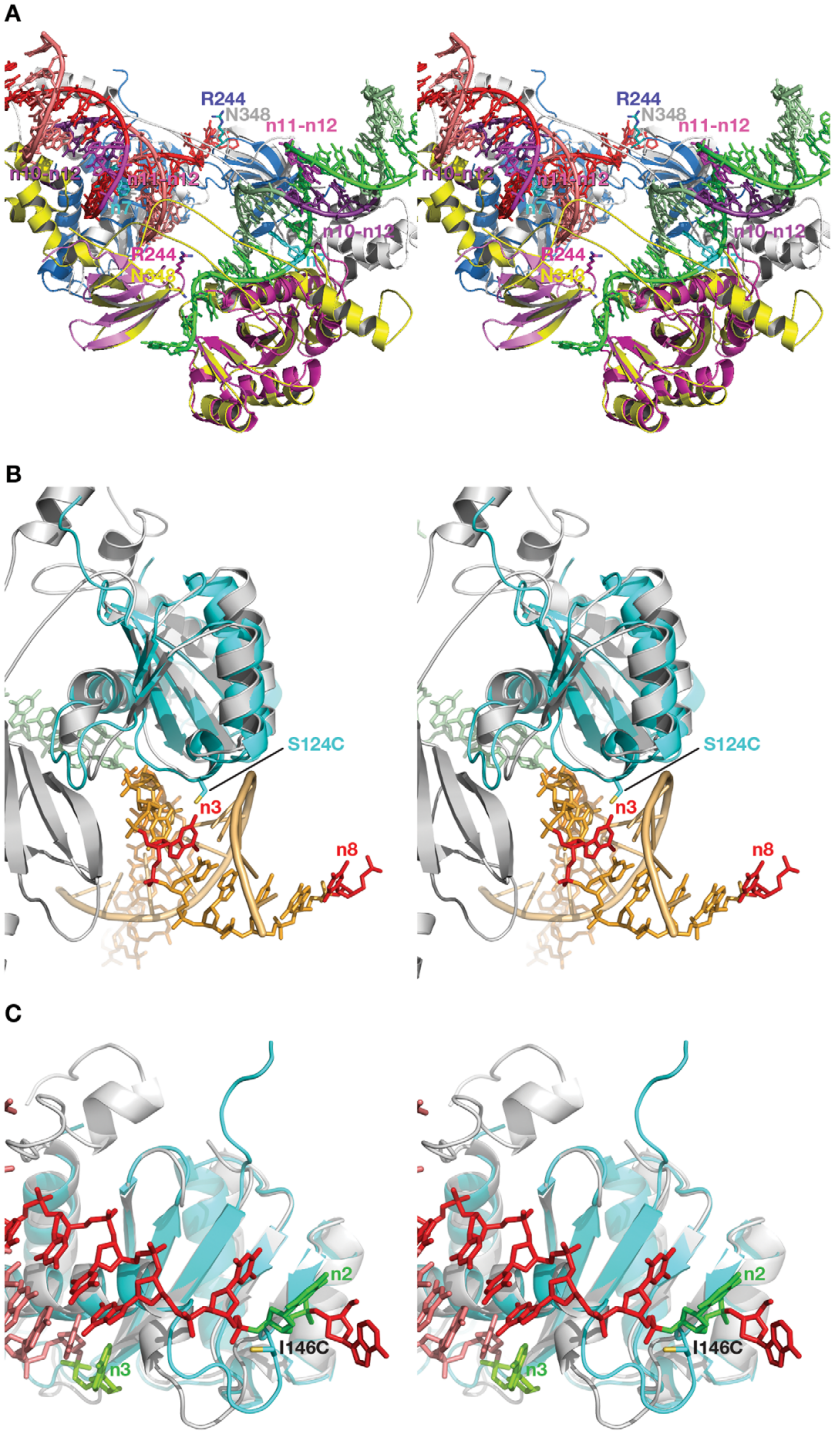
Structural interpretation of crosslinking data for ASV IN. (A) Superposition of a section of the DNA complex of PFV IN (cartoon) containing the two viral DNAs (sticks) with the individual CCD and CTD domains of ASV IN. Individual protomers of the PFV IN tetramer are colored gray and yellow, with Asn348 shown as sticks in corresponding colors. CCDs of ASV IN are magenta and blue and one CTD of ASV is shown in magenta, with Arg244 shown in sticks in corresponding colors. The linker region of PFV is shown in yellow. Nucleotides 10–12 of the non-cleaved viral DNA strand are shown in dark violet, nucleotides 11 and 12 of the cleaved viral DNA strand are shown in magenta, nucleotide 7 of the non-cleaved viral DNA strand is shown in cyan. (B) Superposition of the CCDs of ASV IN (blue) onto PFV IN (gray). The viral DNA after the integration step is shown in green and the host target DNA in orange. Nucleotides 3 and 8 on the host DNA are red and the substituted residue S124C is shown as sticks. (C) An analogous superposition, with viral DNA in red (non-cleaved strand) and pink (processed strand). A flexible loop contains the substituted residue I146C. Nucleotide 2 on the non-cleaved strand and the first nucleotide on the 3′-end of the cleaved strand are shown in green.

For the CTD, none of the individual contacts revealed in ASV and HIV-1 IN proteins by crosslinking or other methods can be correlated with those observed in the crystal structures of PFV IN-DNA complexes (**Figure 6**). Our crosslinking results with ASV IN show contacts of Arg244 to both strands of viral DNA at positions 10–12. However, in the PFV intasome structure [6], the equivalent residue, Asn348, is separated from the corresponding positions on DNA by the linker regions that connect the CCD with the NTD and CTD (**Figure 10A**). We note that while not seen in the PFV intasome structure, CTD interactions with the trans viral DNA remain a possibility and could be accomplished with minor movement of the domain. Results of Gao *et al.* [19], indicate that residues Ser230 and Glu246 of HIV-1IN interact with bases 1 and 7 of the non-cleaved strand of viral DNA, respectively. Crosslinking experiments based on the electron microscopy model obtained by Michel *et al.* [50] provided evidence for contact between Lys266 in HIV-1 IN and nucleotides 6–7 in the non-cleaved strand of viral DNA. These results are not in agreement with the HIV-1 model of Krishnan *et al.* [7], which was derived from the PFV crystal structure. Contact between the CTD of HIV-1 IN and the base of thymidine 6 of the non-cleaved strand of viral DNA as reported by Esposito *et al.* [14], faces the same problems as the contact of nucleotide 7 with residue 246 [19], as the linker regions separate the protein and DNA in the PFV intasome.

Residues Leu234, Arg262, Arg263 and Arg269 in the CTD of HIV-1 IN, which have been shown to interact with DNA by modeling and/or experimental studies, were also implicated as interacting with DNA by several mass-spectrometry and mutagenesis studies (**Figure 6**). Residues that are structurally equivalent to Arg262 in HIV-1 IN are Ile366 and Ser262 in PFV and ASV INs, respectively. Because of the different sizes of the side chains of these residues in the three INs, analogous contacts cannot be made with PFV IN, and for ASV IN this seems problematic. Similarly, the capability of Leu234 and Arg263 in HIV-1 IN to contact DNA appears to correlate with the presence of arginine at structurally equivalent positions in ASV and MuLV IN proteins. However, HIV-1 IN Arg269 and PFV IN Ser373 both interact with DNA. The segment containing Ser373 is located at the very end of the visible CTD of PFV IN, and the flexibility of this part of the protein may facilitate interaction with DNA.

Heuer *et al.* [15] showed that the azidophenacyl photocrosslinker, attached to unique phosphorothioate located between nucleotides 6 and 7 of the cleaved strand of viral DNA, could be crosslinked to the peptide comprising residues 247–270 of HIV-1 IN. While some residues from the corresponding range in PFV IN are within reach of equivalent nucleotides 6 and 7 in the crystal structure, the specific residues in HIV-1 IN that are involved in these interactions are unknown.

#### Interactions between DNA and the CCD

Much more information regarding the sites of contact with DNA is available for the CCDs of various INs. Out of twenty-seven individual residues and 7 peptide ranges identified in 50 experimental data points that were analyzed and presented in **Figures 4** **,** **5** as making contact between the CCD and DNA, thirty-seven IN-DNA contacts corresponded to residues analogous with those observed to interact with DNA in the crystal structure of the PFV intasome.

Our photocrosslinking data indicate that S124C of ASV IN makes contact with the third nucleotide of the cleaved strand of target DNA, and a minor contact with nucleotide 8 of the same strand (**Figure 10B**). In the crystal structure of the PFV intasome the analogous residue makes contacts with nucleotides 3 on the cleaved and 6 on the non-cleaved strands of the target DNA (shown in red in **Figure 10B**, they correspond to nucleotides 3 and 3, respectively, in the numbering system used here). The nucleotide corresponding to nucleotide 8 on host DNA complexed to ASV IN (minor contact) is not visible in the structure of the PFV intasome due to the mobility of the ends of the host DNA in the absence of contacts with the protein. This crosslink might be attributed to the flexibility of the photocrosslinking tether combined with mobility of the ends of host DNA (see Materials and Methods).

Photo- and chemical crosslinking data for I146C of ASV IN identified nucleotide 3 of the cleaved strand of viral DNA as the point of contact. Contact between I146C and nucleotide 2 of the non-cleaved strand of viral DNA was also detected by chemical crosslinking (**Tables 2** **,** **3**). In MuLV, the structural equivalent of this residue is Cys209. Photo- and chemical-crosslinking experiments on MuLV by Vera et al. [29] confirmed the involvement of this residue in the interactions with the viral end of DNA in the active site area. Cys209 in MuLV IN is reported to make contact with nucleotide 1 on the non-cleaved strand of viral DNA (**Figure 4**). The corresponding residue in PFV IN, Thr210, also contacts the base of nucleotide 3, as in ASV IN, but in the non-cleaved DNA strand. All chemical crosslinks involving the ASV I146C derivative are maintained with the bases of the corresponding nucleotides. The contacts between Thr210 and DNA in PFV IN are localized in the minor groove between two strands; therefore the data from ASV IN correlate reasonably well with the PFV structure (**Figure 10C**). Residue 146 in ASV IN and the corresponding residues in HIV-1 and PFV INs are located within the active site flexible loop, which has been shown to adopt multiple conformations in different IN structures with various inhibitor, substrate, and pH/buffer conditions. The tip of this loop can move up to 7 Å under conditions that do not alter the overall three-dimensional structure of the CCD. In the PFV intasome, this loop is inserted between the ends of the complementary strands of viral DNA (**Figure 10C**). Therefore, if a similar position is assumed by the ASV loop when complexed with viral DNA, 146C would be able to interact with nucleotides on both strands.

Photo- and chemical crosslinking data for CCD-DNA contacts have been reported by several other groups. The contact for the HIV-1 residue Lys159 reported by Jenkins et al. [13] is with A3 nucleotide at the 3′-end of the processed strand. This amino acid is equivalent to Lys228 in PFV IN, and it interacts with the phosphate backbone between the nucleotides 3 and 4. The crosslinking observed in [13] between HIV-1 Lys159 and N7 of the base of A3 requires some adjustment of the orientation of A3 base, as seen in the PFV intasome structure.

The results of S-S crosslinking [28] of both blunt and processed DNA substrates and the results of photocrosslinking [14] of blunt DNA substrates to HIV-1 IN Q148 implicate two neighboring nucleotides of the non-cleaved strand of viral DNA, #2 and #1, respectively, for interaction. Although these nucleotides are found in the crystal structure of PFV IN in the vicinity of S217 (analogous to Q148 in HIV-1 IN), their bases, modified for crosslinking experiments, point away from the side chain of S217. As suggested by Krishnan et al. [7], such discrepancies can be attributed to the experimental setup (blunt *vs.* processed substrates) or to conformational mobility of the crosslinker.

Several amino acid residues of HIV-1 IN were reported by Alian et al. [27] to be involved in crosslinking, but these results do not match the IN-DNA contacts found in the PFV intasome structure for the corresponding pairs. There was a very low correlation of crosslinking data for HIV-1 residues 143, 160, and 164 using processed DNA with the model of HIV-1, which is derived from the PFV intasome structure [7]. Nucleotide A1 of the non-cleaved strand of viral DNA identified by crosslinking to interact with all HIV-1 IN three residues (**Figure 4**) cannot reach the corresponding residues in the crystal structure of PFV IN. Only one contact was detected between HIV-1 Y143 and nucleotide A1 when a blunt ended substrate was used [27]. The same contact was identified by Esposito *et al.* in photocrosslinking experiments with blunt DNA substrates [14]. Alian *et al.* [27] suggested that the loop comprising HIV-1 residues 160–164 comes in close proximity to the 5′-end of the non-cleaved strand of viral DNA only during strand transfer. This hypothesis is inconsistent with the HIV-1 IN model [7]; as Lys 160 lies within contact range of G8 and quite far from the integration center, HIV-1 Y143 is not listed as a possible contact with viral end DNA by Krishnan *et al.* [7] but is positioned in close proximity to processed target DNA nucleotides (#1, 2, 3, −1, −2, and −3) closest to the integration site. It should be noted that, under some conditions, DTNB activation can produce nonspecific crosslinks [19].

Gao *et al.* detected contacts between HIV-1 I191C and two nucleotides, 1 and 7 of non-processed viral DNA by S-S crosslinking [19]. In the PFV intasome structure [6], the amide of V260 (I191 in HIV-1 IN) is located 4.5 Å away from the phosphate of nucleotide #7 of the non-cleaved strand of viral DNA, which is reasonable if the length of the thiopropyl linker is taken into account.

While the photocrosslinking experiments in which interactions between specific modified nucleotides and HIV-1 IN [15] in most cases do not provide exact localization of the contact sites on the IN protein, comparison of the relative positions of identified peptides (49–69, 51–64, 139–152, and 158–198) and DNA show good correlation for 11 out of 13 reported crosslinking contacts when compared to the PFV intasome structure [7], the ASV IN two-domain structure (PDB code1C0M) [7] superimposed on the corresponding domains of the PFV intasome, and the model of the HIV-1 intasome [7]. Some of these peptides have been targeted from multiple locations on DNA. For example, HIV-1 peptide 49–69 comes into close proximity to the viral processed DNA (phosphate between C4 and G5, G5 base [15]), non-processed viral DNA (A1 base, and the phosphate between G4 and C5 [15]), and non-cleaved strand of target DNA (backbone of G1, G2, and C(−2), G(−3) [15]). The latter contacts are located on the opposite sides of the same strand of target DNA from the integration site (a similar spatial relationship is illustrated in **Figure 1** for ASV IN substrate) and are made with residues from two IN monomers in the model of HIV-1 IN [7] Introduction of the photoactivatable nucleotide analogs I-dU and I-dC into positions 3 of the cleavable strand and 1 and 2 of non-cleavable strand of blunt viral DNA substrates resulted in the crosslinks with CCD, although the exact positions in the protein were not elucidated [15]. Nucleotides in these positions are also found to be in close proximity to the active site of the CCD in the PFV intasome [7].

Mutagenesis experiments carried out by Chen et al. [21] on HIV-1 IN provided a list of residues (V54, V72, T124, T125, S153, K156, E157, K160, G193, 54–57) likely to be important for DNA binding and substrate specificity. Circular dichroism, fluorescence, and NMR experiments involving a synthetic analog of α4 helix of HIV-1 CCD and U5 LTR end [51] revealed that the HIV-1 IN residues E152, S153, N155, K156, and K159 were likely to make contact with DNA. Protease mapping with HIV-1 IN [49] assigned a similar role to the residues K111, K136, K159, E138, K185, K186, and K188, and mass spectrometry footprinting experiments [48] indicated that K159 and K160 are involved DNA interactions. The corresponding residues in the PFV IN-DNA complex structure are within range to establish contacts with target or viral DNAs. However, the PFV equivalents of some residues in HIV-1 IN implicated in DNA binding in these experiments (e.g. 161, 162, 171, 172, 197 and 201 and peptides 128–130, 163–165), are not in a suitable range to contact DNA in the PFV intasome. Several positions in the fragment comprising residues 207–219, shown to interact with DNA by protease mapping [49] and mass spectrometry [48], belong to the linker region between the CCD and CTD. This region differs in length in HIV-1, ASV, and PFV INs and exhibits little sequence homology. The HIV-1 IN model built by Krishnan *et al.* [7] allows for the residues from this fragment to maintain contacts with non-cleaved strand of viral DNA (**Figures 4** **,** **5**), correlating with the mapping data listed above.

Mutagenesis experiment by Esposito *et al.* [14] indicated that nucleotides 3 ,4, 12, and 13 of the cleaved strand of viral DNA and nucleotide 2 of the non-cleaved strand participate in CCD-DNA interactions. The contacts of the nucleotides 2, 3,and 4 are in good agreement with the model of the HIV-1 intasome and structural data from PFV IN. Similarly, the loop comprising residues 207–209 of HIV-1 IN is in close proximity to nucleotides 12 and 13 of the cleaved strand. While the mutagenesis results [14] do not contradict the structural data, they do not locate the contact residues in the protein. In contrast, our S-S crosslinking data identify both counterparts in the ASV IN-DNA interactions. For example, results with the I146C derivative of ASV IN implicate this residue in interactions with nucleotide 3 of the cleaved strand and nucleotide 2 of the non-cleaved strand of viral DNA.

In conclusion, the high degree of correlation between the structural and biochemical data on IN-DNA contacts in the CCD indicate that the mode of binding DNA to this domain is highly conserved in PFV, HIV-1, and ASV INs. Differences in protein structure and composition may explain the lack of correspondence in details of DNA binding by the NTD and CTD of PFV in the crystal structure of the intasome, when compared with data obtained from analysis of crosslinking and other experiments performed with ASV and HIV-1 IN proteins. The presence of an additional domain at the N terminus of PFV IN (the NED) certainly sets it apart from the other two retroviral IN proteins. In addition, variations in length and sequence of the linker regions between the NTD and CCD, and the CCD and CTD, suggests that residues at different positions in these domains could have been selected to perform analogous functions during the course of evolution of these viruses. On the other hand, depending on the concentration, IN proteins can exist in a variety of multimers in solution (dimers, tetramers, and higher forms), each of which might interact with DNA in unique ways during the assembly of a functional intasome. Such interactions may be detected in biochemical experiments, but not represented in the intasome crystal. Furthermore, the same amino acid in individual subunits might make different contacts with DNA in one or more of these multimers. We note that the NTDs and CTDs of only two of the four component subunits are visible in the crystal of the PFV intasome, and it is unknown if or how these domains in the other two subunits might interact with DNA. Additional crystal structures, including those of other retroviral intasomes, could help to resolve some of these issues. However, until we understand more about the dynamic properties of IN, and the conformational changes that accompany intasome assembly, it will be important to keep all of these factors in mind when interpreting both structural and biochemical data.

## Materials and Methods

### Photocrosslinkers

Heterobifunctional photoactivatable thiol-specific reagents, a carbene-generating N-bromoacetyl-N′-{2,3-dihydroxy-3-[3-(3-(trifluoromethyl)diazirin-3-yl)phenyl]propionyl} ethylenediamine (BATDHP) [17] and nitrene-generating azidophenylthiophtalimide (APTP) from Sigma [32] were used. Photocrosslinking reagents were prepared as 10–20 mM stock solutions in DMSO and stored in the dark at −20°C for no longer than 30 days. These reagents were coupled to the SH- group of the engineered Cys-containing IN derivatives. Amino-reactive photocrosslinking reagent N-hydroxysuccinimidyl-3-[3-(trifluoromethyl)diazirin-3-yl]benzoate was used for modification of NH_2_-derivatized thymidines in DNA substrates to complement our data obtained with Cys-substituted modified proteins.

### Thiol modification

Modification and crosslinking procedures with IN were performed as previously described [17], [18]. The IN proteins were modified with the photocrosslinking reagents via a single Cys residue. 50 µL of 30 µM solutions of IN were treated with 5 mM DTT on ice for 30 min to reduce the SH group. DTT was then removed by gel filtration using Sephadex G50 Centrisep desalting columns (from Princeton Separations, Adelphia, NJ) in buffer 1 (40 mM HEPES pH 6.5, 500 mM NaCl, 5% glycerol). The reduced IN was allowed to react with 10-fold molar excess of a photoreagent in dark vials on ice for 12 hr after raising the pH of reaction mixtures from 6.5 to 7.8 by addition of 1 M Tris-HCl pH 8.0. The appropriate amounts were extrapolated for small volume reactions (20–100 µl) from test-titration of a 100 ml mixture without protein and DNA. Excess photocrosslinking reagent was removed by gel filtration with buffer 1. All subsequent manipulations were carried out under reduced light levels.

### Mass spectrometry

Mass spectrometry, performed at the Fox Chase Cancer Center Shared Facility, was used to determine the number and position(s) of modifications to ensure that Cys residues in the Zn-coordinating motif of the NTD were not modified with crosslinking reagents. IN modified with either BATDHP or APTP was digested with trypsin. Tryptic peptides containing modifications were identified by matrix-assisted laser desorption time-of-flight (MALDI-TOF) spectrometry.

### DNA substrates

Amino-derivatized and non-modified DNA oligonucleotides synthesized using the phosphoroamidite method with subsequent PAGE purification were obtained from commercial sources (Oligos, Etc.). Oligonucleotides were tagged by 5′ labeling with γ^32^P-ATP using T4 polynucleotide kinase (T4 PNK) obtained from Boehringer Mannheim.

The following sequences were prepared:

1 5′ GCTGTTGAATACCATCTAATCGTGTCGGGTCTCGTACTGCGGAA 3′

2 5′ TCCGCAGTACGAGACCCG 3′

3 5′ AATGTAGTCTTATGCAATAGC 3′

3′ 5′ AATGTAGTCTTATGCAATACTC 3′

3f 5′ ATTGTAGTCTTATGCAATACTC 3′

4 5′ GCTATTGCATAAGACTACAACACGATTAGATGGTATTCAACAAGC 3′

4′ 5′ GAGTATTGCATAAGACTACATT 3′

4f 5′ GAGTATTGCATAAGACTACAAT 3′

NH2-3.8 5′ AATGTAG**T**CTTATGCAATACTC 3′

NH2-3.11 5′ AATGTAGTCT**T**ATGCAATACTC 3′

NH2-4.12 5′ GAGTATTGCA**T**AAGACTACATT 3′

where amino-modified nucleotides are shown underlined and in bold.

DNA strands 1–4 were mixed in equal concentrations and annealed to prepare the Y-mer substrate; strands 3′ and 4′ were mixed in equal concentrations and annealed to prepare the linear substrate. Oligonucleotides 3f and 4f were used for preparation of frayed-end substrates with the appropriate modified complementary strands. Amino-modified oligonucleotides were used to introduce the NHS-[3-(3-(trifluoromethyl)diazirine-3-yl]benzoate photoreagent by a procedure similar to modification of IN, except the reducing step.

For chemical crosslinking, oligonucleotides with thiol-modified adenosines and guanidines were prepared similarly to the method of Erlandson et al. [52] (See **Methods S1** for details). Oligonucleotide SH 4.3-P carried a mercaptopropanol phosphate ester -O**_3_**P-O-(CH**_2_**)**_3_**-SH in place of scissile phosphate. In SH 4.3-M the 3′ terminal desoxyribose was substituted with N-mercaptoethyl morpholine. Modified positions below are bolded and underlined; numbering is as in **Figure 1**. For description of synthetic pathways and structures, see **Methods S1**.

SH3.1,7 5′ **A**ATGTA**G**TCTTATGCAATACTC 3′

SH3.1,12 5′ **A**ATGTAGTCTT**A**TGCAATACTC 3′

SH3.2 5′ A**A**TGTAGTCTTATGCAATACTC 3′

SH3.12 5′ AATGTAGTCTT**A**TGCAATACTC 3′

SH4.13 5′ GAGTATTGC**A**TAAGACTACATT 3′

SH4.11 5′ GAGTATTGCAT**A**AGACTACATT 3′

SH4.3 5′ GAGTATTGCATAAGACTAC**A**TT 3′

SH4.3-P 5′ GAGTATTGCATAAGACTAC**A** 3′

SH4.3-M 5′ GAGTATTGCATAAGACTAC**A** 3′

### Photocrosslinking

10 µM IN and 0.05 µM DNA substrate (5′-labeled with γ^32^P ATP at one of the component oligonucleotides) were incubated in buffer 2 (40 mM HEPES pH 6.5, 150 mM NaCl, 5% glycerol) for 15 min at 0°C and then UV-irradiated with a hand-held lamp placed 1 cm away from the samples on ice for 15 min using a glass plate as additional filter (cutoff 315 nm). Non-reducing denaturing PAGE was used to separate crosslinked IN from the non-crosslinked protein, as well as to remove any DNA that was not crosslinked. The products were visualized and quantified with a PhosphorImager (Storm 860 from Molecular Dynamics, Inc., Sunnyvale, CA). The efficiency of crosslinking was calculated as the percent of radioactivity in the IN-DNA bands relative to the total amount of radioactivity in the lane. As an excess of IN protein was used, and both the DNA and IN were present at concentrations significantly higher than the IN-DNA binding constant, all DNA is assumed to be bound to the enzyme. The negative control samples were obtained by UV-irradiation of reaction mixtures with non-modified INs and by analyzing non-irradiated samples.

### Localization of the preferred sites of crosslinking

Localization of the preferred sites of IN photocrosslinking to different DNA substrates under various conditions was performed using Cel 1 “Surveyor” endonuclease from Transgenomics, Inc. (Rockville, MD) [34]–[36], [53]. Samples of the UV-crosslinked IN-DNA complexes were prepared and 2–3 µL aliquots were used to analyze the crosslinking efficiency by PAGE and PhosphorImager. The reaction mixtures were extracted twice by phenol-chloroform. Aqueous fractions that contained non-crosslinked DNA were pooled together, precipitated with ethanol and saved as a negative control for Cel1 analysis. Phenol/chloroform fractions containing covalent IN-DNA complexes were pooled together, divided in half, and one half was treated with 10 mM NaIO_4_ (30 min. at room temperature), while the other half was left untreated. Both phenol-extracted samples were ethanol-precipitated and dissolved in 20 microliters of water. We used non-treated ^32^P-labeled DNA substrates, as well as crosslinking reaction mixtures and crosslinking reaction mixtures after treatment with 10 mM NaIO_4_ as controls for each Cel1 assay. The Cel1 assays were performed according to manufacturer's instructions. The reactions were terminated by adding the formamide gel loading buffer and heating to 90°C. Reaction mixtures were separated by Urea-PAGE using 6% and 20% gels and analyzed with PhosphorImager.

### Chemical crosslinking

Reducing denaturing PAGE was used to break the covalent linkage between DNA and IN in order to confirm that the DNA was in fact crosslinked by a disulfide linkage. Crosslinking reactions were performed by mixing 25 µM IN with corresponding concentration of DNA to produce a desired ratio of IN-DNA in 40 mM HEPES pH 6.5, 150 mM NaCl, 1 mM EDTA and 5% glycerol. After 1 hr preincubation on ice, the pH of the reaction mixture was adjusted to 7.8 by addition of 1 M Tris-HCl pH 8.0 and then left on ice for 10–15 hr to allow crosslinking. PAGE gel analysis with Coomassie staining was used to separate and quantify the products of reactions by densitometry.

Crosslinking experiments with IN derivatives that contained Cys substitutions in catalytic residues D64 and E157 of ASV IN were performed essentially as described above, with minor modifications. The processed, recessed linear DNA substrate (**Figure 1**) was used with the “processed” strand comprising either SH4.3-M or SH4.3-P.

The oligonucleotides were mixed in equimolar quantities and annealed prior to reduction with 100 mM β-ME on ice for 12 hr. The excess of reducing agent was removed by gel filtration on Centrisep spin-columns in 150 mM NaCl. IN was concentrated to ∼5–6 mg/ml in Buffer 1. The concentrated protein was treated with β-mercaptoethanol on ice for 12 hr for reduction of the surface Cys. The excess of reducing agent was removed by gel filtration into Buffer 1. The DNA was then added to a protein solution for a final molar ratio of protein to DNA of 2∶1 or 1∶1. The complex was incubated on ice for 30 min in Buffer 1 before adjustment of NaCl concentration to 250–300 mM and pH to 7.5. The reactions were carried out with and without 10 mM MgCl_2_.

Alternatively, for the catalytic Cys derivatives, S-S bond formation was facilitated with DTNB as in [27]. 1 mM DTNB was added to each of the reduced oligonucleotide substrates and the mixture was incubated for 2 hr at room temperature. Excess DTNB was also removed by gel filtration in 20 mM Tris-HCl pH 8.0, 150 mM NaCl. IN proteins were prepared as above in Buffer 1. 50 µM DNA was added to 50 µM IN protein at either 2∶1 or 1∶1 molar ratio and incubated on ice for 10 min, diluted 1.5-fold with 20 mM Tris-HCl pH 8.0, 150 mM NaCl. Reactions were initiated by adding MnCl_2_ to a final concentration of 10 mM. After overnight incubation on ice, the yields of crosslinking were determined by PAGE after overnight incubation on ice.

## Supporting Information

Figure S1 **Disintegration reactions of the modified IN proteins with Y-mer substrate.** A) Schematic depicting integrase-catalyzed disintegration and joining reactions of the Y-mer substrate superimposed on a generic tetramer model for integrase (left). The liberated viral DNA (right) is the same DNA as seen in a pre-cleaved end, with the cleaved portion of strand 4 indicated by “4-”. B) Disintegration activity of WT and ASV IN derivative proteins before and after modification with photocrosslinking reagents [54]. Upper band on each gel represents the 44-mer product of reaction, lower bands correspond to the 19-mer substrate (5′ ^32^P-labeled Y-mer strand 2*). Due to increased exposure times of the four gels on the right, the contaminating 18-mer fragments of the substrate are also visible. Reaction times were 0–1200 s; increasing time is indicated by the wedge above gel lanes. “noXL” stands for no IN modification with photocrosslinker.
(TIF)

Figure S2 **Strand specificity of photocrosslinking of modified Cys residues in substituted derivatives of ASV IN.** A sample of gels accompanied by bar graphs with the photocrosslinking yields (%) presented. The position of modified Cys residues in the IN derivatives is noted above the bar graph. The crosslinker used is also listed above the graph (noXL stands for no IN modification with photocrosslinker). DNA strands in the Ymer substrate are labeled according to **Figure S1**, and the labeled strands are indicated below each graph. Brackets on the right show the bands corresponding to IN-DNA adducts and free DNA.
(TIF)

Figure S3 **Comparison of the efficiency of photocrosslinking of APTP- and BATDHP-modified ASV IN derivatives to Y-mer (Y3,Y4) and linear (L3, L4) DNA substrates.** Labeling is as in **Figure S2**.
(TIF)

Figure S4 **Comparison of UV photocrosslinking yields of wild type ASV IN to dsDNA substrates modified with diazirine photocrosslinkers.** Positions of the modified bases are bolded and the conserved A is underlined.
(TIF)

Figure S5 **A sample of Coomassie-stained gels with S-S crosslinked IN-DNA complexes.** The substrates are labeled above the lanes. Y stands for Y-mer DNA, L for linear; letters are followed by strand numbers, thiol modified positions are shown after dash. Bands corresponding to crosslinked complexes are marked by arrows. The negative controls are marked with a dash above the left-most lane of each gel. 1IN and 2IN with arrowheads designate monomeric and dimeric IN bands, respectively. The stemmed arrows point to IN-DNA adduct bands. Molecular weight marker lanes are not shown, since the monomer (lower) and dimer (upper) strong bands provide internal calibration.
(TIF)

Methods S1 **A more detailed description of the materials and methods utilized in this work.**
(PDF)

Results S1 **A more detailed analysis of the enzymatic activity of IN and of the chemical crosslinking of modified DNA substrates to ASV IN.**
(PDF)
