# Supplementary figures and images for: Localization of ASV Integrase-DNA Contacts by Site-Directed Crosslinking and their Structural Analysis
Source: PLoS One. 2011 Dec 1;6(12):e27751. doi: 10.1371/journal.pone.0027751 (PMC3228729; doi:10.1371/journal.pone.0027751)

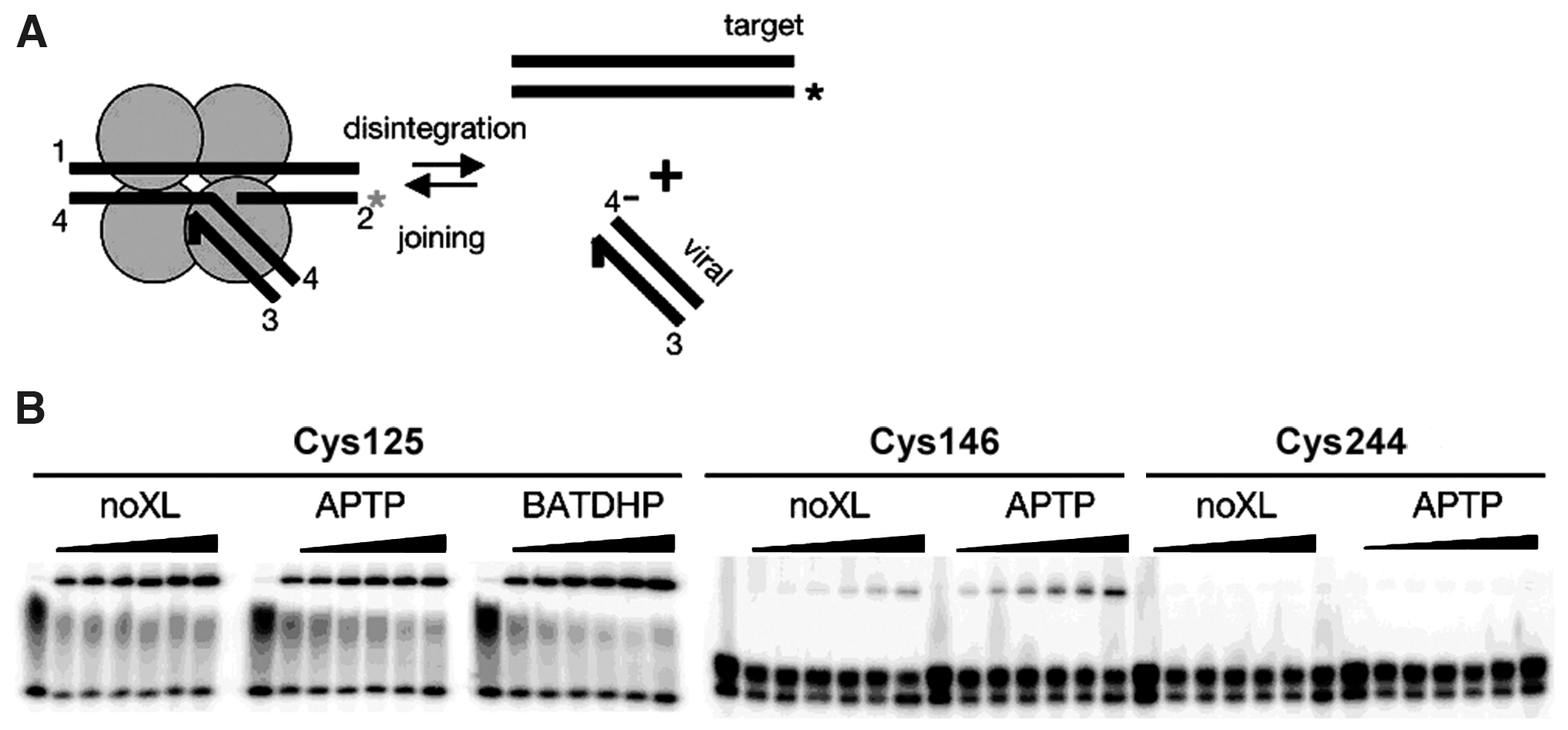

Supplement: Figure S1 — Disintegration reactions of the modified IN proteins with Y-mer substrate. A) Schematic depicting integrase-catalyzed disintegration and joining reactions of the Y-mer substrate superimposed on a generic tetramer model for integrase (left). The liberated viral DNA (right) is the same DNA as seen in a pre-cleaved end, with the cleaved portion of strand 4 indicated by “4-”. B) Disintegration activity of WT and ASV IN derivative proteins before and after modification with photocrosslinking reagents [54]. Upper band on each gel represents the 44-mer product of reaction, lower bands correspond to the 19-mer substrate (5′ 32P-labeled Y-mer strand 2*). Due to increased exposure times of the four gels on the right, the contaminating 18-mer fragments of the substrate are also visible. Reaction times were 0–1200 s; increasing time is indicated by the wedge above gel lanes. “noXL” stands for no IN modification with photocrosslinker. (TIF) [file pone.0027751.s001.tif]

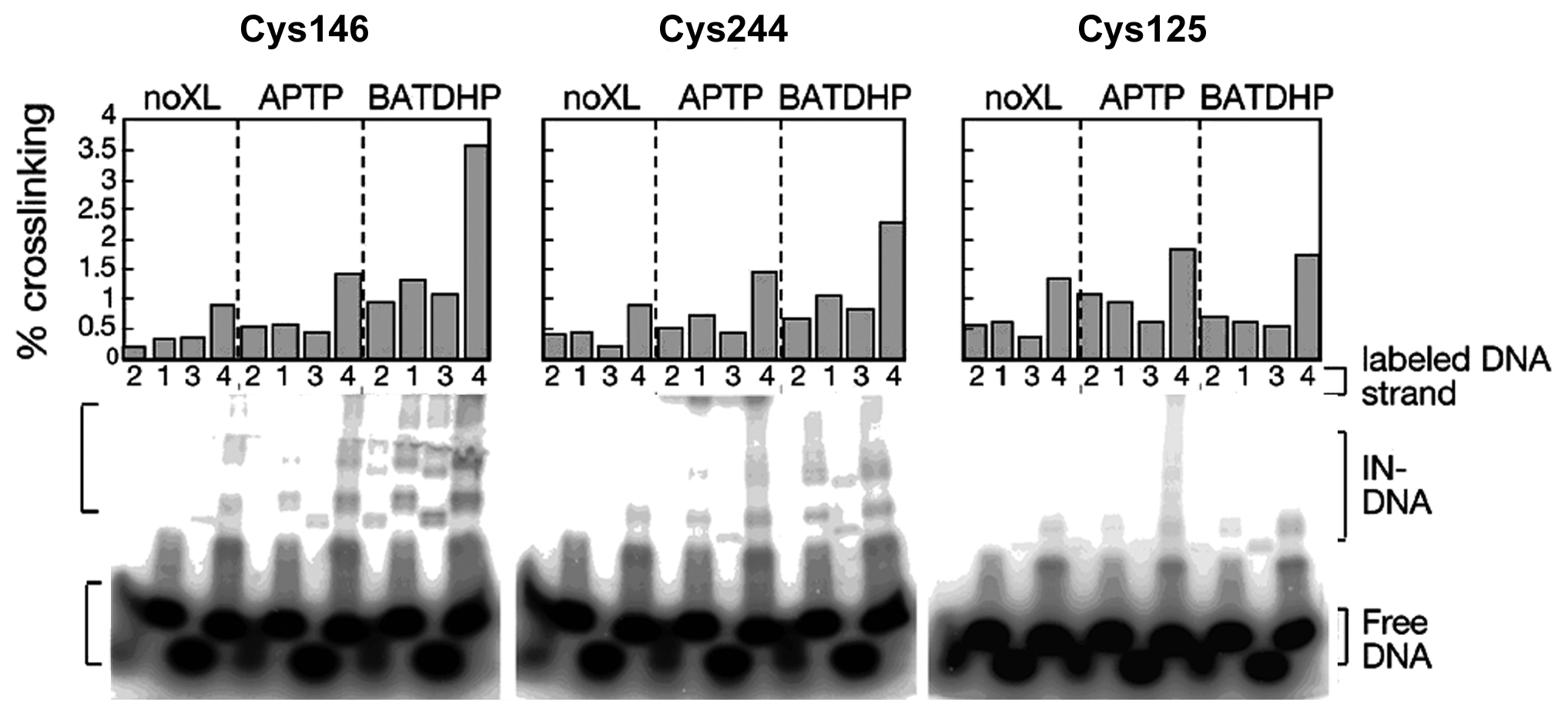

Supplement: Figure S2 — Strand specificity of photocrosslinking of modified Cys residues in substituted derivatives of ASV IN. A sample of gels accompanied by bar graphs with the photocrosslinking yields (%) presented. The position of modified Cys residues in the IN derivatives is noted above the bar graph. The crosslinker used is also listed above the graph (noXL stands for no IN modification with photocrosslinker). DNA strands in the Ymer substrate are labeled according to Figure S1, and the labeled strands are indicated below each graph. Brackets on the right show the bands corresponding to IN-DNA adducts and free DNA. (TIF) [file pone.0027751.s002.tif]

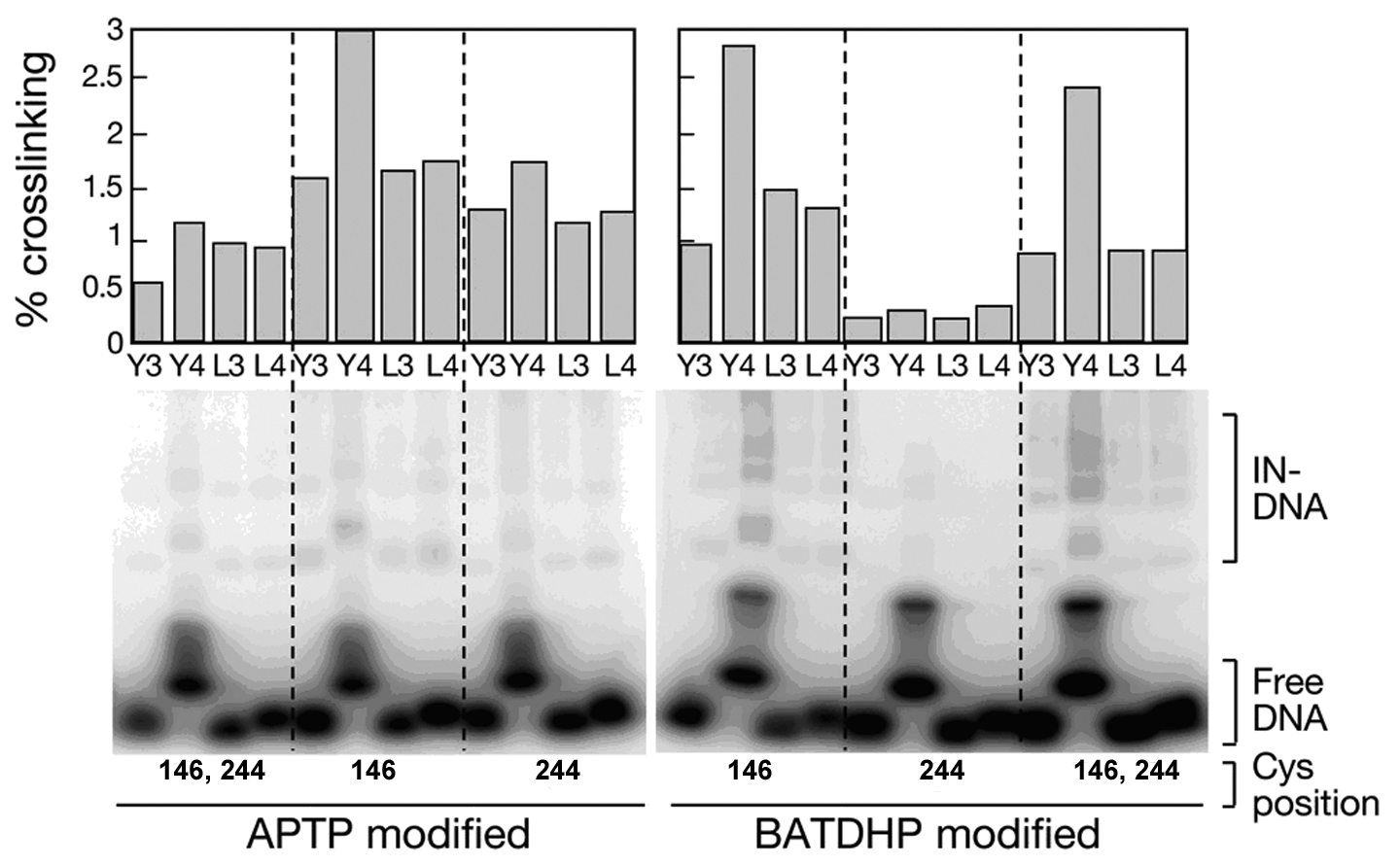

Supplement: Figure S3 — Comparison of the efficiency of photocrosslinking of APTP- and BATDHP-modified ASV IN derivatives to Y-mer (Y3,Y4) and linear (L3, L4) DNA substrates. Labeling is as in Figure S2. (TIF) [file pone.0027751.s003.tif]

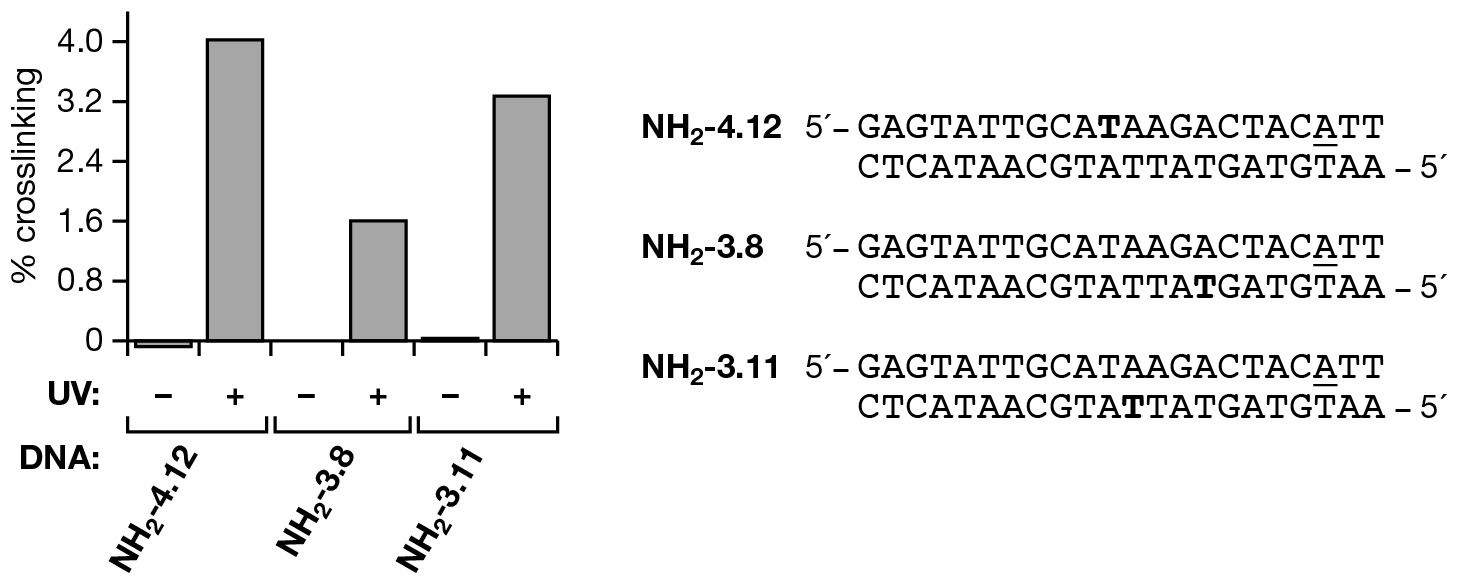

Supplement: Figure S4 — Comparison of UV photocrosslinking yields of wild type ASV IN to dsDNA substrates modified with diazirine photocrosslinkers. Positions of the modified bases are bolded and the conserved A is underlined. (TIF) [file pone.0027751.s004.tif]

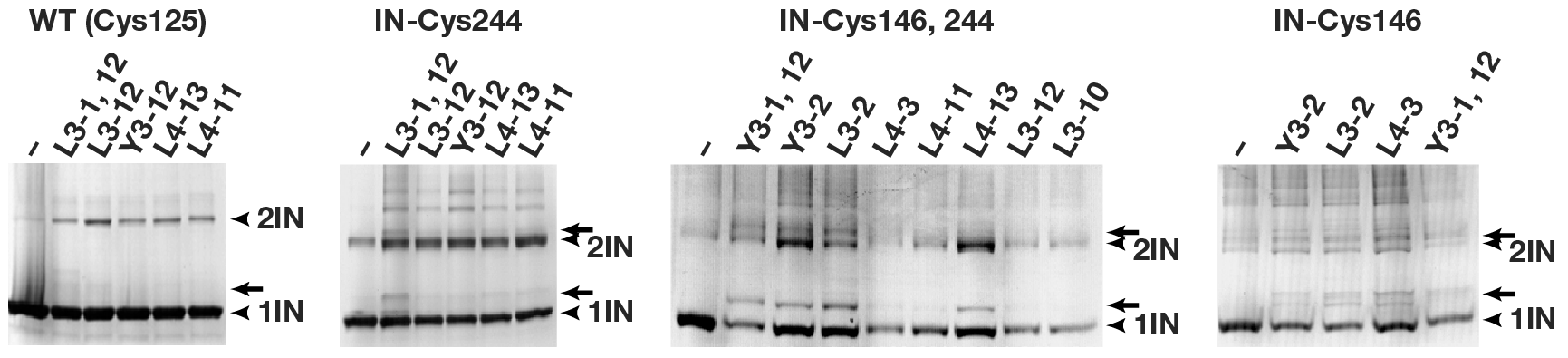

Supplement: Figure S5 — A sample of Coomassie-stained gels with S-S crosslinked IN-DNA complexes. The substrates are labeled above the lanes. Y stands for Y-mer DNA, L for linear; letters are followed by strand numbers, thiol modified positions are shown after dash. Bands corresponding to crosslinked complexes are marked by arrows. The negative controls are marked with a dash above the left-most lane of each gel. 1IN and 2IN with arrowheads designate monomeric and dimeric IN bands, respectively. The stemmed arrows point to IN-DNA adduct bands. Molecular weight marker lanes are not shown, since the monomer (lower) and dimer (upper) strong bands provide internal calibration. (TIF) [file pone.0027751.s005.tif]
