# Supplementary material for: Localization of ASV Integrase-DNA Contacts by Site-Directed Crosslinking and their Structural Analysis
Source: PLoS One. 2011 Dec 1;6(12):e27751. doi: 10.1371/journal.pone.0027751 (PMC3228729; doi:10.1371/journal.pone.0027751)
Supplement: Methods S1 — A more detailed description of the materials and methods utilized in this work. (PDF) [file pone.0027751.s006.pdf]

Supporting Materials and Methods

Photocrosslinking to DNA substrates

The ASV IN derivatives were modified by coupling with two photoactivatable thiol-specific compounds, a carbene-generating compound, N-bromoacetyl-N'-{2,3-dihydroxy-3-[3-(3-(trifluoromethyl)diazirin-3-yl)phenyl]propionyl}ethylenediamine (BATDHP) [1] or a nitrene-generating compound, azidophenacylthiopyridine (APTP) [2]. Carbenes are among the most reactive moieties known. They are capable of insertion into any chemical bond present in a biomolecule, including aliphatic chains and aromatic rings. Having nanosecond half-lifetimes, carbenes rapidly form covalent bonds with neighboring atoms. Although electrophilic, carbenes are so highly reactive that in the absence of strong nucleophiles they attack even the C-H bonds (over 80% crosslinking to cyclohexane) [3,4]. The high level of reactivity of carbenes with buffer components usually precludes high yields of crosslinked products but their low specificity makes them ideal for identification and localization of members and parts of affinity complex. Nitrenes such as those generated from the azide reagent, APTP, are less reactive and tend to undergo intramolecular rearrangements that lead to even less reactive products. They crosslink primarily to nucleophiles such as amino groups and, in a non-nucleophilic environment, can remain active for periods of up to several minutes. This makes them less reliable for detection of close interactions since selective crosslinking may preferentially occur to a relatively distant nucleophilic group that is only occasionally in the vicinity of the crosslinker. The efficiency of crosslinking with nitrenes is thus higher, but there is a possibility of bias towards interactions with nucleophilic groups that are not relevant to the complex under study [4].

Oligonucleotide synthesis with 8-amino-3,4-dithiaoctyl tether at N^2 of dG

The oligonucleotides were prepared on commercial dC-cpg (40-50 μmol/g) using a standard synthesizer protocol for generation of the 21-base sequence at the 3'-end. The 5'-DMT protected phosphoramidite [5] derived from O^6-(2-*p*-nitrophenylethyl)-2-fluoro-2'-deoxyinosine was coupled manually to the 5'-end of the oligonucleotide bound to the support [6]. A typical 2-μmole synthesis utilized 20 mg (21 μmol) of the phosphoramidite and 150 μL of 0.5 M 4,5-dicyanoimidazole in acetonitrile for 16 h at rt.; yield was estimated from the recovery of DMT cation after deprotection. End capping with acetic anhydride was omitted after the manual coupling step [6], and the support-bound oligonucleotide was directly oxidized (1 M *tert*-butyl hydroperoxide in dichloromethane, 30 s) and returned to the synthesizer for addition of the remaining 8 residues by the standard automated synthesis procedure. After removal of the 5'-DMT protecting group, the linker was coupled to the support-bound oligonucleotide using a

modification of the procedure of Erlanson et al. [5]. The support-bound oligonucleotide was treated with 45 mg (178 µmol) of 3,3'-dithiobis(propylamine) dihydrochloride [7], which was prepared as described for the 4-carbon homolog [8] 60 µL (600 µmol) triethylamine and 100 µL H_2O for 16 h at room temperature. In the course of preparing O^6-(2-*p*-nitrophenylethyl)-2-fluoro-2'-deoxyinosine, we observed cleavage of the nitrophenylethyl protecting group in the presence of wet *tert*-butyl ammonium fluoride. After reaction with dithiobis(propylamine), addition of concentrated NH_4OH (1.5 mL) containing 20 µmol *tert*-butyl ammonium fluoride to the beads and solution, followed by heating at 60 ºC for 3 days, resulted in complete deblocking of the oligonucleotide; this procedure avoided the DBU/formamide cleavage step and accompanying formylation [5] of the free amino group of the tether. After filtration, the oligonucleotide solution was dialyzed against 0.1 M triethylammonium acetate buffer (pH 6.0) overnight to remove excess amines. The oligonucleotide was purified by HPLC on a Hamilton PRP-1 column (7 µm, 10 x 250 mm) eluted at 3 mL/min with a linear gradient of acetonitrile in 0.1 M $(NH_4)_2HCO_3$ buffer (pH 7.5) that increased the acetonitrile concentration from 0 to 17.5% over 20 min; t_r 16.3 min.

Synthesis of the 3' modified viral end substrates for crosslinking to the active site IN derivatives. Oligonucleotides 5'-GAGTATTGCATAAGACTAC-**A***-3' where **A*** represents the following structures:

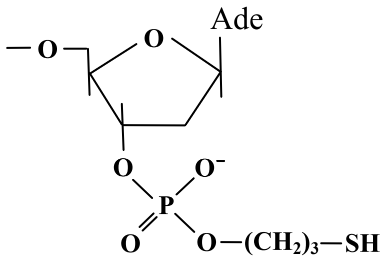

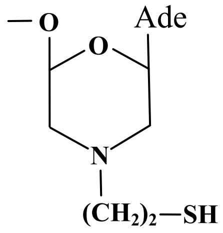

were synthesized following a standard protocol using modified solid support of the following structure: CPG-700-LCAA-NH-CO-$(CH_2)_2$-COO-$(CH_2)_2$-SS-$(CH_2)_3$-O-DMTr

For deblocking a standard mixture of ammonia and methylamine also contained 50mM DTT for disulfide cleavage.

Schematics of nucleotide synthesis:

3’-end N-mercaptoethyl morpholine-adenosine was synthesized according to Stirchak et al. [9]. To complete the morpholine ring we used cystamine acetate, $(NH_2\text{-}(CH_2)_2\text{-}SS\text{-}(CH_2)_2\text{-}NH_2)Ac_2$, for amino component. The disulfide was cleaved by DTT to yield corresponding mercaptane:

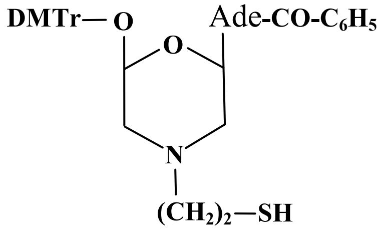

that was then attached to modified solid support. The support modification consisted of mercaptoethanol treatment to achieve corresponding polymer-mercaptane, followed by activation with dipyridyl disulfide.

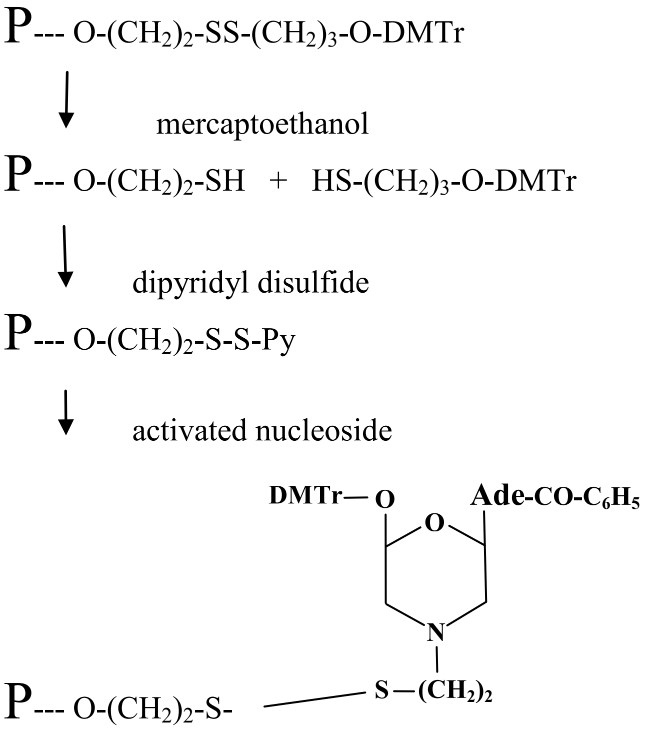

Isolation of the synthesized oligonucleotide was performed as described above. Both oligonucleotides were purified by RP-HPLC and their structures confirmed by mass-spectrometry (MALDI-TOF).

References

1. Peletskaya EN, Boyer PL, Kogon AA, Clark P, Kroth H, Sayer JM, Jerina DM, Hughes SH (2001) Cross-linking of the fingers subdomain of human immunodeficiency virus type 1 reverse transcriptase to template-primer. J Virol 75: 9435-9445.

2. Moreland RB, Smith PK, Fujimoto EK, Dockter ME (1982) Synthesis and characterization of N-(4-azidophenylthio)phthalimide: A cleavable, photoactivable crosslinking reagent that reacts with sulfhydryl groups. Anal Biochem 121: 321-326.

3. Weber PJ, Beck-Sickinger AG (1997) Comparison of the photochemical behavior of four different photoactivatable probes. J Pept Res 49: 375-383.

4. Tate JJ, Persinger J, Bartholomew B (1998) Survey of four different photoreactive moieties for DNA photoaffinity labeling of yeast RNA polymerase III transcription complexes. Nucleic Acids Res 26: 1421-1426.

5. Erlanson DA, Chen L, Verdine GL (1993) Allosteric effects on enzyme action in disulfide-crosslinked DNA. J Am Chem Soc 115: 12583-12584.

6. Custer L, Zajc B, Sayer JM, Cullinane C, Phillips DR, Cheh AM, Jerina DM, Bohr VA, Mazur SJ (1999) Stereospecific differences in repair by human cell extracts of synthesized oligonucleotides containing trans-opened 7,8,9, 10-tetrahydrobenzo[a]pyrene 7,8-diol 9,10-epoxide N2-dG adduct stereoisomers located within the human K-ras codon 12 sequence. Biochemistry 38: 569-581.

7. Evans BJ, Doi JT, Musker WK (1990) Kinetics of the aqueous periodate oxidation of aliphatic disulfides and thioethers. J Org Chem 55: 2580-2586.

8. Dirscherl W, Weingarten FW (1951) Synthese von Homologen des Cystamins. Justus Liebigs Ann Chem 574: 131-139.

9. Stirchak EP, Summerton JE, Weller DD (1989) Uncharged stereoregular nucleic acid analogs: 2. Morpholino nucleoside oligomers with carbamate internucleoside linkages. Nucleic Acids Res 17: 6129-6141.
