# Supplementary material for: Localization of ASV Integrase-DNA Contacts by Site-Directed Crosslinking and their Structural Analysis
Source: PLoS One. 2011 Dec 1;6(12):e27751. doi: 10.1371/journal.pone.0027751 (PMC3228729; doi:10.1371/journal.pone.0027751)
Supplement: Results S1 — A more detailed analysis of the enzymatic activity of IN and of the chemical crosslinking of modified DNA substrates to ASV IN. (PDF) [file pone.0027751.s007.pdf]

Supporting Results

Modification of Cys residues does not compromise enzymatic activity of ASV IN

Modification of wild type (WT) ASV IN (Cys125) with cysteine-specific photo-crosslinker caused only minor decrease in the rate of the disintegration reaction. Similar results have been obtained for both I146C and R244C proteins (see **Table 1**). Modification of wild type (WT) ASV IN (Cys125) with cysteine-specific photo-crosslinker caused only minor decrease in the rate of the disintegration reaction. These proteins retained 20% and 2% of the WT activity prior to modification, respectively, but suffered no additional loss of activity after the modification; disintegration activity of the protein with a modified I146C is even slightly higher than before modification (**Fig. S1**). Similar results have been obtained for processing and joining activities for both I146C and R244C containing proteins (see **Table 1**).

Photocrosslinking studies were performed with APTP and BATDHP-modified Cys- and WT ASV IN proteins, complexed with Y-mer DNA substrates, representing integration intermediate, that were separately radioactively labeled at the 5' end of each of the four component strands (labeled 1-4 in **Fig. S2**). Because an excess of IN was used and both the DNA and IN were present at concentrations significantly higher than the IN-DNA binding constant, all the DNA was assumed to be bound to the enzyme. UV-irradiated reaction mixtures with non-modified IN and non-irradiated reaction mixtures of modified samples served as negative controls. As expected, 340-370 nm UV does not cause any significant crosslinking of DNA to IN, here demonstrated by the results with non-modified IN proteins (**Fig. S2**, lanes noXL).

As shown in Figure S2, the IN derivative that carries Cys at position 146 in the active site loop gave the highest yields of crosslinking, almost twice as high as the other two proteins tested. Significant differences in crosslinking among the three proteins tested confirm that the Zn-binding Cys residues in the NTD, present in all cases, were not modified and/or did not contribute significantly to the IN-DNA crosslinking. WT IN crosslinking (Cys at position 125) was largely non-specific as demonstrated by the low crosslinking yields for both modified and non-modified proteins. With all the IN proteins analyzed, oligonucleotide 4 (see **Fig. S2**) was preferred for crosslinking, although the derivative with Cys in the CTD (R244C) has significantly less pronounced preference for strand 4. This strand of the bound DNA is analogous to the newly joined viral and host DNA strand, catalyzed by IN. The diazirine

photocrosslinker BATDHP shows higher crosslinking efficiency than the azide-based APTP, possibly because it is 8 Å longer. Since guanidines are the best (if not the only) nucleophilic target for nitrenes to crosslink to, lower azide efficiency can be interpreted either as absence of G bases in the section of DNA within reach of APTP, or that the overall distance to DNA was too long for APTP to crosslink but still reachable for BATDHP.

We also compared photocrosslinking efficiencies of Y-mer and linear viral DNA substrates (see **Fig. S3**). For the IN derivatives with the following modified Cys residues: I146C, I146C/R244C, and R244C, strand 4 of the Y-mer substrate was found to be the most likely target for crosslinking. Two strands of the short linear dsDNA substrate representing viral end (L3 and L4) showed no clear preference for interaction with modified INs. Overall crosslinking efficiency to Cys at position 244 was low. This may be explained by reduced DNA-binding capacity of the IN derivative with an R244C substitution.

Chemical crosslinking of modified DNA substrates to ASV IN

The chemical crosslinking experiments were designed so that the reaction (S-S crosslinking) involved a particular chemical group on both biomolecules (IN and DNA). The thiol groups have only a minimal reactivity to other chemical moieties including buffer components and water. As there are no other targets in the reaction mixture, this type of chemical crosslinking is efficient. Unlike photoactivatable reagents the chemical crosslinkers are reactive during the whole time of an experiment. This means crosslinking can occur whenever the modified elements are close enough for interaction, which does not necessarily require that these elements be appropriately complexed. However, in the publication in which an SH- group on DNA was crosslinked to the thumb of HIV-1 RT, crosslinking was specific and depended on proper alignment of the SH-groups on the protein and the nucleic acid [1]. We used similar methods to obtain IN-DNA complexes and analyze localization of the contact sites.

Reference

1. Huang H, Chopra R, Verdine GL, Harrison SC (1998) Structure of a covalently trapped catalytic complex of HIV-1 reverse transcriptase: implications for drug resistance. Science 282: 1669-1675.
